# Deletion of mitochondrial calcium uniporter enhances calcium signals by slowing calcium clearance and triggers adaptive transcriptomic remodeling

**DOI:** 10.1016/j.jbc.2026.111473

**Published:** 2026-04-17

**Authors:** Rajesh Bhardwaj, Abdull J. Massri, Gary S. Bird, Anant B. Parekh

**Affiliations:** 1 Molecular & Cellular Biology Laboratory, National Institute of Environmental Health Sciences, National Institutes of Health, Durham, North Carolina, USA; 2 Integrative Bioinformatics Support Group, National Institute of Environmental Health Sciences, National Institutes of Health, Durham, North Carolina, USA

**Keywords:** Ca^2+^ release-activated Ca^2+^ (CRAC) channel, calcium homeostasis, cytosolic calcium clearance, mitochondrial calcium uniporter (MCU), plasma membrane Ca^2+^ ATPase (PMCA), store-operated calcium entry (SOCE), transcriptomics

## Abstract

Mitochondrial Ca^2+^ uptake *via* the mitochondrial Ca^2+^ uniporter (MCU) following store-operated Ca^2+^ entry supports cellular bioenergetics, yet how mitochondria shape store-operated Ca^2+^ entry and cytosolic Ca^2+^ signaling remains incompletely understood. Combining gene deletion and functional Ca^2+^ imaging techniques with a rigorous transcriptomic filter, we find larger cytosolic Ca^2+^ signals in CRISPR/Cas9-generated *Mcu* KO cells. This increase arises primarily from slower cytosolic Ca^2+^ clearance rather than increased store-operated Ca^2+^ release-activated Ca^2+^ (CRAC) channel activity. Compensatory upregulation of cytosolic Ca^2+^ regulators, such as the plasma membrane Ca^2+^ ATPase pump that extrudes excess cytosolic Ca^2+^, is insufficient to restore normal Ca^2+^ homeostasis. Reexpression of WT MCU restored the cytosolic Ca^2+^ dynamics but a channel pore-dead MCU mutant did not. Deletion of *Mcu* resulted in major alterations in the transcriptome and reexpression of the protein significantly restored 15% of more than 200 common genes that showed differential expression in two independent KO clones. Our results identify a set of candidate MCU-dependent genes that may contribute to the regulation of cellular Ca^2+^ signaling, and show how cytosolic Ca^2+^ signals can be enhanced in the absence of MCU without an increase in Ca^2+^ release-activated Ca^2+^ channel activity.

Mitochondria have well-established functions in cellular energy production, intracellular Ca^2+^ signaling, crosstalk with other organelles through tethering and in orchestrating apoptosis (1, 2). Dysregulated mitochondrial function is tightly involved in the development of pathological processes, including neurodegeneration (3).

Through their ability to buffer cytosolic Ca^2+^, mitochondria provide a mechanism to couple cell excitation with energetic demand. Ca^2+^ uptake into the mitochondrial matrix activates the rate-limiting enzymes pyruvate dehydrogenase, α-ketoglutarate dehydrogenase, and isocitrate dehydrogenase to stimulate metabolic flux through the Krebs cycle (4). This leads to increased ATP production (5) that is required for activating numerous Ca^2+^-dependent responses, as well as for exporting Ca^2+^ from the cytosol by primary active transport after stimulus termination.

Mitochondrial Ca^2+^ uptake across the inner mitochondrial membrane is driven by the large electric gradient of ∼ -180 mV, and which is established by H^+^ expulsion through the electron transport chain (6). The inner mitochondrial membrane is populated with mitochondrial Ca^2+^ uniporter (MCU) proteins, pore-forming tetrameric Ca^2+^-selective ion channels which, when open, provide low conductance pathways for Ca^2+^ to enter the matrix (7, 8). MCU is an integral part of a larger multimeric protein complex that contains regulators of ion channel activity (9, 10, 11).

Multiple studies have demonstrated that mitochondria are tethered to other organelles including the endoplasmic reticulum (ER), lysosomes, and plasma membrane (12). Tethering has been best studied at ER-mitochondria contact sites. At such locations, MCU detects high levels of local Ca^2+^ that build up rapidly near open InsP_3_-gated Ca^2+^ release channels on the ER (13, 14). Exposure to high local Ca^2+^ enables rapid mitochondrial Ca^2+^ uptake through the MCU. However, mitochondria also respond to bulk rises in cytosolic Ca^2+^, which can be achieved following opening of plasma membrane Ca^2+^ channels (14).

In nonexcitable cells, store-operated Ca^2+^ release-activated Ca^2+^ (CRAC) channels provide a major route for Ca^2+^ entry. These channels are opened by depletion of the Ca^2+^ content of the ER (15). Store depletion is sensed by the single-pass transmembrane proteins STIM1 and STIM2, through canonical EF hand domains that face the lumen of the store (16, 17). Ca^2+^ unbinding from the EF hands as ER Ca^2+^ falls results in a conformational change that unmasks cytosolic domains, and which leads to exposure of the cytosolic CRAC channel activation domain (18). STIM then forms oligomers which migrate across the ER to specialized regions just below the plasma membrane (19). Here, they bind and open the plasma membrane protein Orai1, the pore-forming subunit of the CRAC channel (20, 21, 22, 23).

Several studies have shown that mitochondrial Ca^2+^ uptake regulates CRAC channel activity (24, 25, 26, 27, 28, 29) by preventing Ca^2+^-dependent slow inactivation of the channels (24, 25, 26, 27). This form of inactivation develops over tens of seconds, requires a rise in bulk cytosolic Ca^2+^ and is driven by a mechanism that is located at least 100 nm from the CRAC channel pore (30, 31). Consistent with this, numerous groups have demonstrated in different cell types that siRNA-based knockdown of the MCU reduces the amplitude of store-operated Ca^2+^ entry (32, 33, 34, 35, 36, 37). The reduction in Ca^2+^ influx varied between 40% and 80%, depending on cell type and the extent of MCU protein knockdown.

Intriguingly, recent studies using CRISPR/Cas9-based knockout (KO) of the *Mcu* gene have provided somewhat different and conflicting results. In *Mcu*^−/−^ cells derived from jurkat T-lymphocytes, RBL-1 and A20 B cells, the cytoplasmic Ca^2+^ signal following store-operated Ca^2+^ entry (SOCE) was larger than in wild-type (WT) cells, although no difference was seen in HEK293 cells (38). Murine T cells with genetic ablation of the *Mcu* were reported to increase store-operated Ca^2+^ influx when compared with WT cells (39). However, in CD4^+^ conventional T cells and in Tregs, SOCE was unaffected by *Mcu* gene ablation (40).

In this study, we have used the CRISPR/Cas9 strategy to generate *Mcu*^−/−^ RBL-2H3 cells, a cell line we have used previously to characterize the role of mitochondrial Ca^2+^ uptake on Ca^2+^-dependent slow inactivation of CRAC channels. We confirm recent studies that have demonstrated enhanced cytosolic Ca^2+^ following SOCE, but we show this is not due to increased store-operated Ca^2+^ influx itself. Instead, plasma membrane Ca^2+^ ATPase (PMCA) activity is unable to cope with the cytosolic Ca^2+^ rise in the *Mcu*^−/−^ cells, resulting in slower Ca^2+^ clearance and therefore a larger cytosolic Ca^2+^ rise following store-operated Ca^2+^ influx. Unexpectedly, our RNA-seq data reveal hundreds of genes are differentially expressed in *Mcu*^−/−^ clones, including several associated with intracellular Ca^2+^ signaling. Re-introduction of the MCU into *Mcu*^−/−^ cells rescued store-operated Ca^2+^ signals, in that the latter now closely mirrored those in WT cells, but did not fully reverse the broad changes in transcriptomics. Our results show that genetic deletion of the MCU gene leads to an apparent increase in SOCE, but the elevated Ca^2+^ is due to reduced Ca^2+^ clearance from the cytosol. Our data also establish a set of genes, several of which regulate cytosolic Ca^2+^ signaling, and whose normal transcriptional profile is dependent on the MCU.

## Results

### MCU is absent following CRISPR/Cas9-dependent deletion of the *Mcu* gene

**Figure 1:**
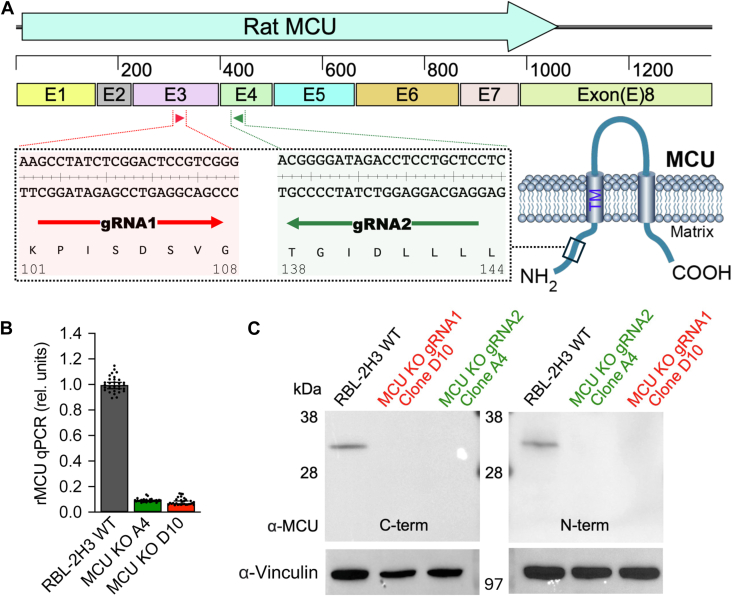
**CRISPR/Cas9-mediated knockout of *Mcu* in RBL-2H3 cells.***A*, exon structure of rat *Mcu* indicating the target sites of guide RNA1 and guide RNA2. *B*, quantitative real-time PCR analysis of *Mcu* mRNA expression in wild-type (WT) RBL-2H3 cells and *Mcu* knockout (KO) clones A4 and D10 (n = 30 wells; 3 biological replicates). Medians (*bars*) with 95% confidence intervals (error *bars*) are displayed on the scatter dot plots. *C*, immunoblot analysis of WT and *Mcu* KO clones A4 and D10 using MCU antibodies raised against either the C terminus or N terminus. Vinculin served as a loading control. Molecular weights are indicated in kilodaltons (kDa). MCU, mitochondrial Ca^2+^ uniporter.

We used a CRISPR/Cas9 strategy to delete the *Mcu* gene in RBL-2H3 cells. Two different guide RNAs (gRNAs) were selected from all possible target sites within exons common to all transcripts, with preference for sequences encoding the N-terminal region, based on the best available combination of on-target and off-target scores (see Experimental procedures). Guide RNA1 targeted a region on exon 3 encoding amino acid residues 101 to 108, whereas guide RNA2 was directed at a region on exon 4 encoding residues 138 to 144 (Fig. 1*A*). Both targeted the cytosolic N terminus of the channel, well before the channel pore, to militate against potential expression of a partially functioning ion channel (Fig. 1*A*). Quantitative real time PCR of two single cell-derived isogenic clones A4 (derived from guide RNA2) and D10 (derived from guide RNA1) showed a strong reduction in *Mcu* mRNA in both (Fig. 1*B*). Western blots using an antibody directed against the C terminus of the MCU confirmed that the protein was absent in both clones, whereas a prominent band of around 35 kDa was seen in the parental RBL cell line (Fig. 1*C*, left hand panel). To rule out the synthesis of a C-terminal truncated MCU protein, we ran western blot with an antibody directed against the N terminus of the MCU. Whereas a clear band of ∼35 kDa was seen in WT cells, no protein was detectable in either clone (Fig. 1*C*, right hand panel). Therefore, both A4 and D10 clones do not express MCU protein.

### Cytosolic Ca^2+^ signals are enhanced following store-operated Ca^2+^ influx in *Mcu*^−/−^ cells

**Figure 2:**
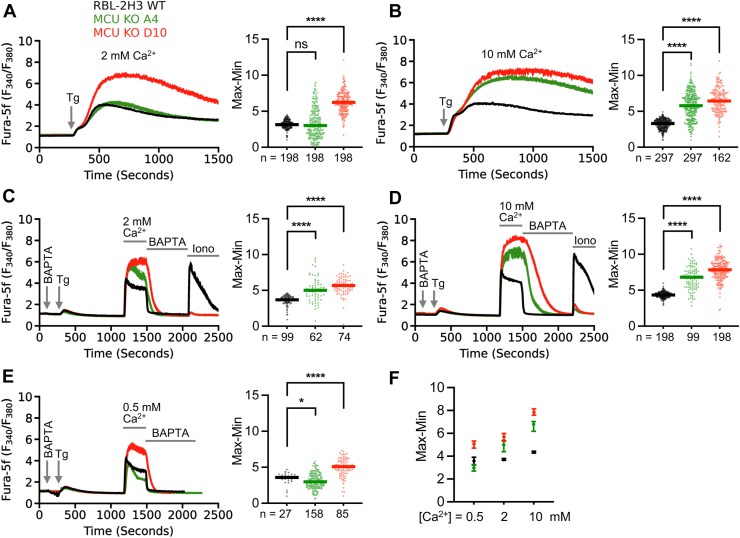
**Loss of MCU enhances the cytosolic Ca^2+^ signal following SOCE.***A*, ratiometric Ca^2+^ imaging traces (average ± standard error of mean) of WT (*black*) RBL-2H3 cells and *Mcu* KO clones A4 (*green*) and D10 (*red*) from single-cell Ca^2+^ imaging experiments. Thapsigargin (2 μM) was applied in extracellular solution containing 2 mM CaCl_2_. Thapsigargin was applied at the *arrow* and maintained throughout. *B*, as in *panel A*, but or 10 mM CaCl_2_ was used instead. For each trace, the value at 240 s (prestimulation) was subtracted from the response at 1200 s (peak response); the resulting max–min values are quantified (n = number of cells) on the *right* of each Ca^2+^ trace. *Solid lines* represent the medians. *C–E*, traces show Ca^2+^ signals in Ca^2+^ add-back assays in WT and *Mcu* KO cells. The solution was exchanged from 2 mM CaCl_2_ to Ca^2+^-free solution containing 100 μM of cell-impermeable Ca^2+^ chelator BAPTA (indicated by BAPTA in each trace), after which cells were exposed to 2 μM thapsigargin (Tg) under these Ca^2+^-free conditions. Reintroduction of external Ca^2+^ was performed with either 2 mM (*C*), 10 mM (*D*) or (*E*) 0.5 mM CaCl_2_. Extracellular Ca^2+^ was removed by switching to BAPTA-containing extracellular Ca^2+^-free solution. Mitochondrial Ca^2+^ release was evoked by application of 10 μM ionomycin (Iono) in Ca^2+^-free buffer, as indicated. Max–Min values were calculated by subtracting the minimum at 1150 s from the maximum at 1485 s (n = number of cells). *F*, comparison of max-min values of Ca^2+^ entry-induced signals from traces in *C*–*E*. Data are presented as medians with 95% confidence intervals (*error bars*). Statistical significance was determined using either Student’s *t* test or the Mann–Whitney test, based on the outcome of the Shapiro–Wilk normality test. Significance levels are denoted as ns (*p*> 0.05), ∗ (*p*≤ 0.05), ∗∗ (*p*≤ 0.01), ∗∗∗ (*p*≤ 0.001), and ∗∗∗∗ (*p*≤ 0.0001). MCU, mitochondrial Ca^2+^ uniporter; SOCE, store-operated Ca^2+^ entry.

To assess whether Ca^2+^ signals following SOCE were altered by the absence of MCU, we measured cytosolic Ca^2+^ following stimulation with thapsigargin. Thapsigargin inhibits sarco-endoplasmic reticulum Ca^2+^ ATPase (SERCA) pump activity, preventing Ca^2+^ store refilling. In the presence of continuous Ca^2+^ leakage from the stores, block of SERCA pumps with thapsigargin results in gradual store depletion and thus activation of SOCE. Exposure to thapsigargin in the continuous presence of 2 mM extracellular Ca^2+^ resulted in a slowly rising cytosolic Ca^2+^ signal that subsequently declined to reach a sustained plateau in WT cells (black trace in Figure 2*A*). Although the average response in A4 cells was not significantly different from WT ones (green trace in Fig. 2*A*), a subset of cells showed elevated responses (Fig. 2*A*, right hand panel). However, a considerably larger Ca^2+^ signal occurred in the D10 clone (red trace in Fig. 2*A*). Averaged data comparing the peak responses are summarized in the right-hand panel of Figure 2*A*. We repeated these experiments with *Mcu* KO clones A4 and D10 in 10 mM extracellular Ca^2+^, expecting quantitatively larger signals but which mirrored the pattern in 2 mM Ca^2+^. Slightly larger Ca^2+^ signals were seen in WT cells (Fig. 2*B*) but the Ca^2+^ signals in both A4 and D10 clones were considerably larger than the corresponding responses in 2 mM Ca^2+^ (Fig. 2*B*).

To separate Ca^2+^ release from SOCE, we used the well-established Ca^2+^ add-back protocol (Fig. 2*C*). WT cells were challenged with thapsigargin in Ca^2+^-free solution to measure Ca^2+^ release from the stores. After 15 min, 2 mM Ca^2+^ was readmitted and cytosolic Ca^2+^ rose quickly due to Ca^2+^ entry through the open store-operated Ca^2+^ channels (Fig. 2*C*). This Ca^2+^ rise was suppressed by CRAC channel blocker BTP2 (Fig. S1). Strikingly different results were obtained with the A4 and D10 clones. Although Ca^2+^ release in response to thapsigargin was comparable between the clones and WT cells (Fig. 2*C*), the cytosolic Ca^2+^ signal upon Ca^2+^ add-back was larger for each clone (Fig. 2*C*). Similar findings were observed when 10 mM Ca^2+^ was readmitted instead (Fig. 2*D*). Again, the cytosolic Ca^2+^ signal upon readmission of 10 mM Ca^2+^ was larger in A4 and D10 clones compared with WT cells. By contrast, the marked difference in the amplitude of cytosolic Ca^2+^ upon Ca^2+^ add-back between WT cells and the A4 clone was considerably less when 0.5 mM extracellular Ca^2+^ was added instead (Fig. 2*E*). The cytosolic Ca^2+^ increase following Ca^2+^ add-back in the D10 clone remained considerably larger than in WT cells. The relationship between extracellular Ca^2+^ and the amplitude of the cytosolic Ca^2+^ signal following Ca^2+^ entry through store-operated Ca^2+^ channels is shown in Figure 2*F*.

The observations that first, the cytosolic Ca^2+^ signal in store-depleted cells upon readmission of 2 or 10 mM external Ca^2+^ was larger in *Mcu*^−/−^ clones compared with WT cells and second, Ca^2+^ signals in 10 mM Ca^2+^ were considerably larger than in 2 mM Ca^2+^ for both *Mcu*^−/−^ cell lines, were surprising. The K_D_ for Ca^2+^ permeation through CRAC channels in patch clamp experiments is ∼0.7 mM (41). This is in close agreement with results from RBL cells extracted from a FlexStation 3-based system using Calcium 6 (EC_50_ of 0.6 mM) or Fura-2QBT (EC_50_ of 0.4 mM) Ca^2+^ indicators (https://www.moleculardevices.com/en/assets/app-note/br/measure-crac-channel-activity-on-flexstation-3-reader). Raising extracellular Ca^2+^ from 2 to 10 mM therefore should have had little effect on the cytosolic Ca^2+^ signal. This was confirmed in experiments on WT cells (Fig. 2*F*) wherein cytosolic Ca^2+^ increased only modestly upon switching from 2 to 10 mM extracellular Ca^2+^. The finding that the corresponding responses in 10 mM Ca^2+^ were considerably larger in *Mcu*^−/−^ cells compared either with 2 mM Ca^2+^ or WT cells in 10 mM Ca^2+^, and at times before Ca^2+^-dependent slow inactivation would have developed (30, 31), suggests either that loss of the MCU affects the pore properties of the CRAC channel, which seems improbable, or has effects on other mechanisms that help determine the size of the Ca^2+^ signal. In the following experiments, we explored the latter possibility.

### Mitochondria are unable to take up Ca^2+^ in *Mcu*^−/−^ cells

We considered the possibility that *Mcu*^−/−^ cells had adapted to the loss of the MCU by expressing a compensatory mechanism that enabled mitochondria to take up Ca^2+^ through another pathway. To test this, we assessed the Ca^2+^ content of mitochondria in WT and *Mcu*^−/−^ clones, using ionomycin to rapidly release Ca^2+^ from the organelle.

In Figure 2, *C* and *D*, the prolonged rise in cytosolic Ca^2+^ due to SOCE should load the mitochondria with Ca^2+^. After 5 min of Ca^2+^ influx, the bathing solution was replaced with Ca^2+^-free solution supplemented with 0.1 mM BAPTA (cell-impermeable Ca^2+^ chelator). Cytosolic Ca^2+^ fell steeply in WT cells (Fig. 2*C*), due to Ca^2+^ removal from the cytosol by **PMCA** pumps and mitochondrial Ca^2+^ buffering. Subsequent stimulation with ionomycin in Ca^2+^-free solution released Ca^2+^ from a thapsigargin-insensitive Ca^2+^ store, resulting in a large cytosolic Ca^2+^ transient (Fig. 2, *C* and *D*). This Ca^2+^ store was composed almost exclusively of mitochondria because ionomycin failed to raise cytosolic Ca^2+^ when applied after exposure to FCCP (carbonyl cyanide 4-(trifluoromethoxy)phenylhydrazone), a protonophore that depolarizes and thereby releases Ca^2+^ from mitochondria into the cytosol (Fig. S2).

Using the same experimental protocol, cytosolic Ca^2+^ fell considerably more slowly in A4 and D10 clones following removal of extracellular Ca^2+^ (Fig. 2, *C* and *D*). Despite the larger cytosolic Ca^2+^ signal upon readmission of 2 mM Ca^2+^ in *Mcu*^−/−^ clones, subsequent challenge with ionomycin failed to increase cytosolic Ca^2+^ at all (Fig. 2*C*). Similar results were seen when 10 mM extracellular Ca^2+^ was used instead (Fig. 2*D*). Importantly, the mitochondrial membrane potential was similar between WT and *Mcu*^−/−^ clones (Fig. S3), indicating that the lack of mitochondrial Ca^2+^ uptake was not due to altered ΔΨm. Therefore, in the absence of MCU, mitochondria are unable to take up cytosolic Ca^2+^. Furthermore, there is no compensatory mitochondrial Ca^2+^ uptake pathway in *Mcu*^−/−^ cells under our conditions.

### *Stim1* and *Orai1* expression in *Mcu*^−/−^ cells

Another explanation for why the cytosolic Ca^2+^ signal following SOCE is enhanced in *Mcu*^−/−^ cells is that STIM1 and ORAI1 expression has increased, resulting in more functional plasma membrane channels. To test this, we compared *Stim1* and *Orai1* expression between WT cells and A4 and D10 clones by quantitative real time PCR. *Stim1* expression was comparable in all three clones (Fig. S4*A*). *Orai1* expression was modestly reduced in A4 clone and was slightly increased in D10 (Fig. S4*B*). These small, opposing differences in *Orai1* mRNA expression levels cannot explain the larger cytosolic Ca^2+^ rise observed following SOCE in both *Mcu*^−/−^ clones.

### Store-operated channel activity is not enhanced in *Mcu*^−/−^ cells

**Figure 3:**
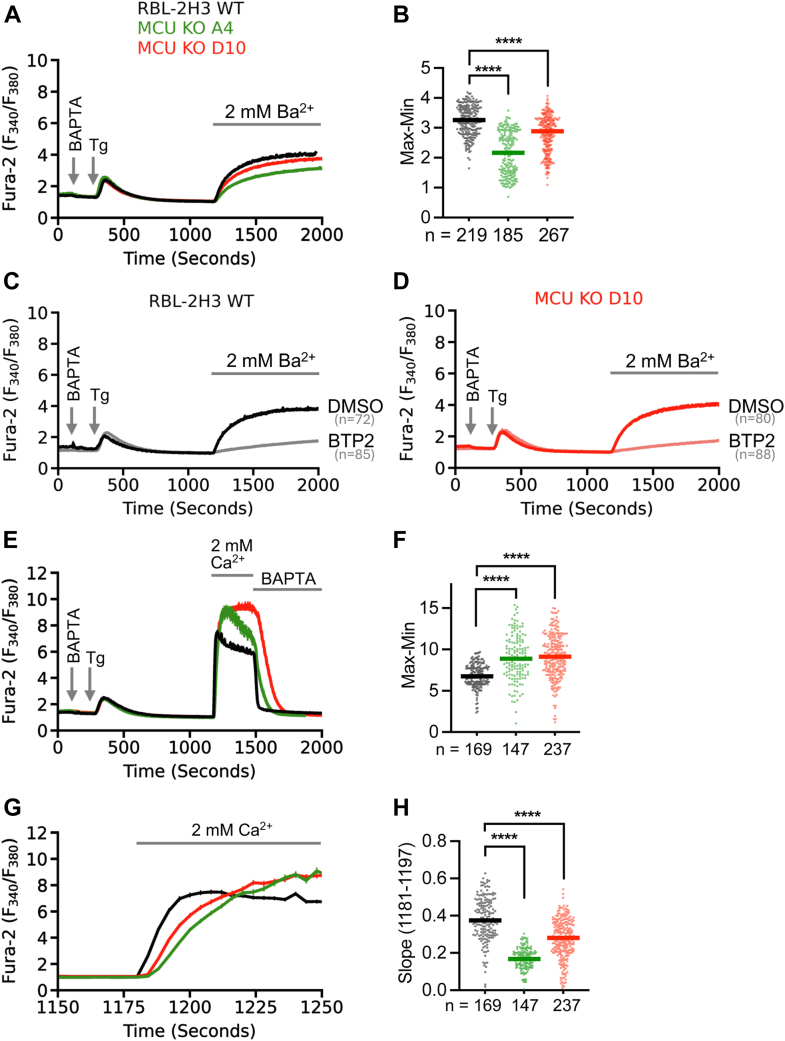
**CRAC channel activity is reduced in *Mcu*^−/−^ cells.***A*, cytosolic Ba^2+^ traces (average ± standard error of mean) measured using ratiometric Fura-2 imaging in WT and *Mcu* KO clones A4 and D10. Exchange from 2 mM Ca^2+^ to Ca^2+^-free solution is indicated by BAPTA (100 μM). Following store depletion with thapsigargin (2 μM) in external Ca^2+^-free solution containing BAPTA, extracellular solution was replaced with Ca^2+^-free solution containing 2 mM Ba^2+^. *B*, quantification of Ba^2+^ entry is presented as max-min (1800 s–1150 s), with *solid lines* representing medians and n = number of cells. *C* and *D*, Ba^2+^ influx after 10 min preincubation with DMSO control or with CRAC channel blocker BTP2 (10 μM) in thapsigargin-treated WT (*C*) and *Mcu* KO D10 cells (*D*). *E*, average (± standard error of mean) traces of cytosolic Ca^2+^ influx upon re-addition of extracellular Ca^2+^ following store depletion in WT and *Mcu* KO clones A4 and D10. *F*, corresponding max-min quantifications of the Ca^2+^ entry phase (1485 s – 1150 s). *G*, magnified view of the Ca^2+^ entry phase from traces in *panel E*. *H*, quantification of the initial rate of rise (slope) of cytosolic Ca^2+^ entry in WT and both A4 and D10 *Mcu* KO clones (n = number of cells). CRAC, Ca^2+^ release-activated Ca^2+^; DMSO, dimethyl sulfoxide; MCU, mitochondrial Ca^2+^ uniporter.

We hypothesized that CRAC channel activity had increased in the *Mcu*^−/−^ clones, which would provide a straightforward explanation for why the cytosolic Ca^2+^ signals were larger following SOCE in the A4 and D10 cell lines. To address this under conditions that closely mimic the preceding cytosolic Ca^2+^ experiments, we took advantage of the fact that Ba^2+^ is an excellent surrogate for Ca^2+^ in permeating CRAC channels (42). Ba^2+^ binds to Fura-2 with a slightly lower affinity than Ca^2+^ but, unlike Ca^2+^, is not transported out of the cytosol by Ca^2+^ ATPase pumps. Ba^2+^ flux therefore provides a reliable indication of CRAC channel activity (42). Following store depletion with thapsigargin in Ca^2+^-free solution, we applied Ca^2+^-free extracellular solution containing 2 mM Ba^2+^. In WT cells, a prominent rise in cytosolic Ba^2+^ occurred (Fig. 3, *A* and *B*), and this was inhibited by the CRAC channel blocker BTP2 (Fig. 3*C*). Ba^2+^ flux into cells from both A4 and D10 clones was significantly reduced compared with WT cells (Fig. 3*A*), with Ba^2+^ entry in cells from the A4 clone showing a higher degree of reduction (Fig. 3, *A* and *B*). As with WT cells, BTP2 blocked the Ba^2+^ flux in *Mcu*^−/−^ cells (Fig. 3*D*). These experiments demonstrate that CRAC channel activity is not increased in *Mcu*^−/−^ cells. If this is indeed true, then a prediction is that the initial rate of rise of cytosolic Ca^2+^ upon readmission of extracellular Ca^2+^ to store-depleted cells should be slower in *Mcu*^−/−^ clones compared with WT cells, despite the larger overall cytosolic Ca^2+^ signal in the former cell lines. The initial rise in cytosolic Ca^2+^ is a better indicator of Ca^2+^ channel activity than the peak cytosolic Ca^2+^ response, because it occurs before Ca^2+^ clearance mechanisms have effectively developed. We found that the rate of rise of cytosolic Ca^2+^ following SOCE was slower in both *Mcu*^−/−^ clones, compared with WT cells (Fig. 3, *E*, *G* and *H*), despite the cytosolic Ca^2+^ peak response being larger in both *Mcu*^−/−^ clones (Fig. 3*F*). A magnified view of the Ca^2+^ entry phase from the experiments in Figure 3*E* is presented in Figure 3*G*. Consistent with lower flux of Ba^2+^ (Fig. 3*B*), *Mcu* KO A4 cells showed a stronger reduction in rate of Ca^2+^ entry as assessed by the slope (Fig. 3, *G* and *H*). The increase in cytosolic Ca^2+^ following SOCE in *Mcu*^−/−^ cells is therefore not a consequence of enhanced CRAC channel activity.

### RNA-seq identifies multiple differentially expressed genes in *Mcu* KO clones

**Figure 4:**
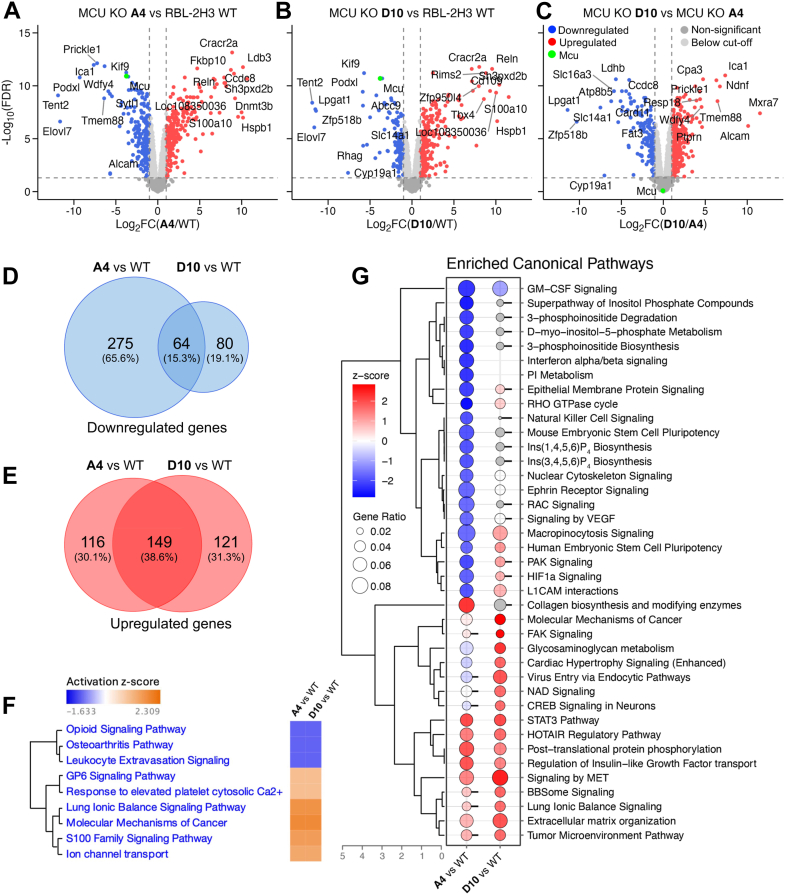
***Mcu* knockout by CRISPR/Cas9 causes widespread and divergent transcriptomic changes across clones generated using distinct guide RNAs.***A* and *B*, volcano plots showing differentially expressed genes (DEGs) in *Mcu*^−/−^ clones A4 (*A*) and D10 (*B*) relative to WT cells. *Red* and *blue dots* represent significantly upregulated and downregulated genes, respectively. *Mcu* is shown as a *green dot*. *Gray dots* indicate unchanged genes, either with below absolute fold change 2.0 cutoff (*light gray*) or having false discovery rate(FDR) higher than 0.05 (*dark gray*). *C*, volcano plot comparing A4 and D10 clones directly. *D* and *E*, Venn diagrams of downregulated genes (*D*) and upregulated genes (*E*) in A4 *versus* WT and D10 *versus* WT, highlighting both overlapping and clone-specific expression changes. *F*, ingenuity pathway analysis (IPA) of the 213 DEGs common to both clones (64 downregulated and 149 upregulated) with a z-score cutoff of 1.0. *G*, bubble plot displays enriched canonical pathways predicted by IPA. The *y*-axis on the right shows enriched pathway names ordered according to hierarchical clustering of their z-scores across two conditions (A4 *versus* WT and D10 *versus* WT), with the corresponding dendrogram displayed on the *left*. The *x*-axis labels the two conditions. Each pathway is represented by two *circles*, one for each condition. Circle size corresponds to the gene ratio for that pathway in the given condition. The fill color of each circle reflects the z-score: *red* for positive z-scores (activation), *blue* for negative z-scores (inhibition), *transparent* for values near zero, and *gray* where the z-score is NA. Threshold of z-score was set to absolute z-score ≥ 1.5. Below cutoff *p* values (*i.e.*, *p* value > 0.05) are indicated by small lines on the *right* of the bubble. MCU, mitochondrial Ca^2+^ uniporter.

Since we were able to rule out changes in CRAC channel activity as the underlying cause for the larger cytosolic Ca^2+^ signals in *Mcu*^−/−^ cells compared with WT counterparts, we hypothesized that other Ca^2+^ signaling pathways might have been compromised by the loss of MCU. To address this directly, we carried out RNA-seq analysis on WT cells and A4 and D10 clones. Both hierarchical clustering dendrogram and multidimensional scaling (MDS) analysis showed tight clustering of WT, A4, and D10 triplicates, with small Euclidean distances on MDS plot (ranging from 0.03–0.09 for WT, 0.04–0.08 for A4, and 0.07–0.20 for D10) indicating minimal variability among replicates, but with clear separation between groups (Fig. S5, *A* and *B*). To our surprise, multiple genes were differentially expressed following knockout of the *Mcu*. The volcano plot in Figure 4*A* shows a large number of genes were upregulated (depicted in red) and downregulated (depicted in blue) in the A4 clone compared with WT cells, while several genes did not change between the two cell lines (shown in gray). Similarly, a remarkable number of genes were differentially expressed in the D10 clone compared with WT cells (Fig. 4*B*).

We compared differentially expressed genes (DEGs) between WT, A4 and D10 clones, and depicted relative comparisons in the Venn diagrams of Figure 4, *D* and *E*. After filtering with a minimum count threshold of 200 in at least one group, 14,364 genes were filtered out and 8890 annotated genes were retained for analysis. Differential gene expression analysis revealed that 339 genes had decreased expression when the A4 cell line was compared with WT cells, but less than half this number of genes were downregulated in D10 clone compared to WT (144 genes). Only 64 of these genes were commonly downregulated in both KO clones (Fig. 4*D*, Table S1), albeit to different degrees. In addition, 265 genes were upregulated in *Mcu* KO clone A4 compared with WT cells, and a similar number of genes were upregulated in clone D10 compared with WT (270 genes). Among these, 149 genes were commonly upregulated in both KO clones (Fig. 4*E*, Table S1). Unexpectedly, 596 genes were differentially expressed between D10 and A4 clones (Fig. 4*C*). Among these 596 DEGs, 24 genes appeared as differentially expressed between D10 and A4 because their upregulation or downregulation relative to WT differed at least 2-fold. In contrast, some genes appeared as DEGs between D10 and A4 because they were regulated in opposite directions relative to WT in each clone. For example, 12 genes (including *Slc16a3*, *Card11*, *Ltbp1*, *Zfp799*, *Ldhb*, and *Mt2a*) that were upregulated in A4 compared to WT were downregulated in D10, whereas eight genes (including *Cpa3*, *Alcam*, and *Mxra7*) that were downregulated in A4 compared to WT were upregulated in D10. Clonal transcriptomic variability can also be noted from Figure 4, *D* and *E*, wherein 275 and 80 genes were exclusively downregulated in clone A4 and D10, respectively. Similarly, 116 genes were exclusively upregulated in clone A4, and 121 genes were upregulated only in *Mcu* KO clone D10. When only the common 64 downregulated and 149 upregulated genes (a total of 213 DEGs between both A4 and D10 clones) were subjected to ingenuity pathway analysis with a z-score cutoff of 1.0, nine pathways were found to be altered (Fig. 4*F*). Six pathways were commonly predicted to be activated, which included GP6 signaling, response to elevated platelet cytosolic Ca^2+^, S100 family signaling pathway, and ion channel transport, whereas three pathways were inactivated. Comparative pathway analysis (z-score cutoff 1.5) of all the 607 DEGs in A4 *versus* WT group and 417 DEGs in D10 *versus* WT revealed that multiple signaling pathways had been affected after knocking out the *Mcu* in both clones, albeit to different extents (Fig. 4*G*). Changes in the interferon alpha/beta signaling, phosphatidylinositol metabolism, nuclear cytoskeleton signaling, ephrin receptor signaling and signaling by vascular endothelial growth factor were all seen in the A4 but not the D10 clone (Fig. 4*G*), despite both showing deletion of the *Mcu* gene. Furthermore, a few of the pathways were even impacted in different directions, inactivated in A4 and activated in D10, such as cardiac hypertrophy signaling and glycosaminoglycan metabolism.

We were intrigued by these substantial differences in gene expression between WT cells and *Mcu* KO clones on the one hand, and between the two clones themselves. We considered various explanations for these findings. One possibility is that the changes in gene expression are a direct consequence of either the loss of MCU itself or of Ca^2+^ import into mitochondria by MCU. Alternatively, the large number of DEGs in the *Mcu*^−/−^ clones could reflect broad changes in cellular transcriptomics by the CRISPR/Cas9 approach. To distinguish between these two possibilities, we expressed MCU in A4 and D10 clones, to see whether the reintroduction of the channel could (i) rescue the Ca^2+^ flux phenotype and (ii) reverse the changes in transcriptomics to more closely mirror WT cells.

### Expression of WT MCU rescues cytosolic Ca^2+^ signaling and the mitochondrial Ca^2+^ pool in *Mcu*^−/−^ cells

**Figure 5:**
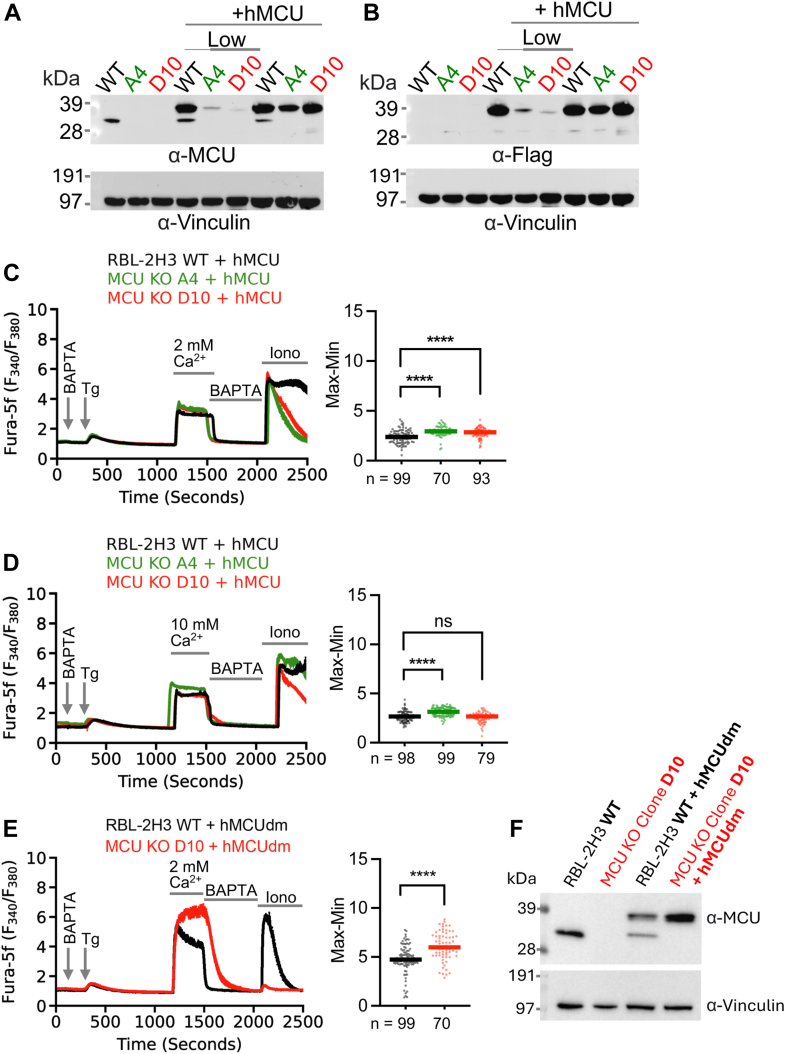
**Re-expression of MCU but not a pore-dead mutant rescues elevated cytosolic Ca^2+^ following SOCE and mitochondrial Ca^2+^ pools in *Mcu*^−/−^ clones.***A* and *B*, MCU (*A*) and FLAG (*B*) immunoblots showing MCU expression in RBL-2H3 WT, *Mcu* KO A4, and *Mcu* KO D10 cells (lanes 1–3), WT, A4, and D10 cells transduced with human *MCU*-FLAG-T2A-copGFP lentiviral construct and sorted for low GFP expression (lanes 4–6) and moderate expression levels (lanes 7–9). Vinculin served as a loading control. Molecular weights are indicated in kilodaltons (kDa). *C* and *D*, cytosolic Ca^2+^ measurements in response to thapsigargin stimulation are shown. Thapsigargin was applied in Ca^2+^-free solution, followed by subsequent readmission of 2 mM (*C*) or 10 mM (*D*) extracellular Ca^2+^. Extracellular Ca^2+^ was then removed (Ca^2+^-free solution containing 100 μM BAPTA), and then 10 μM ionomycin (Iono) in Ca^2+^-free solution was applied. Corresponding max-min (1485 s–1150 s) quantifications of the cytosolic Ca^2+^ signals following Ca^2+^ entry are shown on the *right*. *E*, cytosolic Ca^2+^ signals following SOCE were measured in WT RBL-2H3 cells and *Mcu* KO clone D10 transduced with a pore-dead human *MCU* D261Q-E264Q double mutant (h*MCU*dm) with same external solution exchanges as in *C* and *D*. *Solid lines* in the quantification panels showing scatter dot plots represent medians (n = number of cells). *F*, MCU immunoblot showing MCU expression in RBL-2H3 WT, MCU KO D10, and hMCUdm-expressing WT and D10 cells. Vinculin was used as a loading control. Molecular weights are indicated in kilodaltons (kDa). MCU, mitochondrial Ca^2+^ uniporter; SOCE, store-operated Ca^2+^ entry.

Human MCU (hMCU) shares 97% sequence identity with rat MCU. Taking advantage of the easier availability of the human construct, we expressed human MCU in WT cells and in *Mcu*^−/−^ A4 and D10 clones using a lentiviral transduction approach. RBL-2H3 cells co-expressing hMCU with a C-terminal FLAG-tag and GFP *via* a T2A ribosomal skipping sequence were sorted by flow cytometry to obtain populations with moderate and comparable GFP expression levels across cell types (WT, A4, and D10). The expression of MCU was verified by western blotting using both MCU and FLAG antibodies (Fig. 5, *A* and *B*; rightmost three lanes). Expression of hMCU was comparable in all three cell lines. Stimulation with thapsigargin in Ca^2+^-free solution evoked similar Ca^2+^ release for all three cell lines (Fig. 5*C*). Readmission of extracellular solution containing either 2 mM or 10 mM Ca^2+^ resulted in a rapid cytosolic Ca^2+^ rise that reached a stable plateau (Fig. 5, *C* and *D*). In contrast to the pronounced differences observed in *Mcu* KO cells (Fig. 2, *C* and *D*), both the initial peak amplitude and plateau size were comparable among all three cell lines re-expressing MCU (Fig. 5, *C* and *D*). Removal of extracellular Ca^2+^ led to a steep decrease in cytosolic Ca^2+^ as Ca^2+^ was exported from the cell by the PMCA pump. Subsequent challenge of WT cells with ionomycin in Ca^2+^-free extracellular solution resulted in a prominent rise in cytosolic Ca^2+^, as Ca^2+^ was released from mitochondria which had been loaded with Ca^2+^ during the preceding SOCE phase (Fig. 5, *C* and *D*). Importantly, in A4 and D10 clones expressing recombinant MCU, mitochondria had been loaded with Ca^2+^ to a similar extent as WT cells (Fig. 5, *C* and *D*), demonstrating expression of a fully functional MCU.

Next, we addressed whether a lower expression amount of reintroduced MCU would also rescue the Ca^2+^ phenotype in KO clones A4 and D10. To achieve this, we flow sorted the reintroduced hMCU cells (A4, and D10) with low expression levels of GFP. However, due to a very homogenous expression, we failed to sort the WT cells reintroduced with hMCU into a low GFP expressing population. The western blot showed the corresponding low expression levels of hMCU in clones A4 and D10, but as expected not for WT (Fig. 5, *A* and *B*; middle three lanes marked low). Consistently, the low expressed hMCU in both clones A4 and D10, rescued the ionomycin-releasable mitochondrial pools as well as normalized the elevated cytosolic Ca^2+^ phenotype, bringing the levels closer to WT cells (Fig. S6, *A*–*I*). The sustained elevation of cytosolic Ca^2+^ observed after 1 h of continuous exposure to extracellular Ca^2+^ in *Mcu* KO D10 RBL-2H3 cells was reduced to WT levels with low expression levels of reintroduced hMCU (Fig. S6, *J* and *K*).

Unlike the case with hMCU, expression of a pore-dead MCU D261Q-E264Q double mutant (hMCUdm) in *Mcu* KO clone D10 failed to reverse the exaggerated cytosolic Ca^2+^ phenotype or the recovery of mitochondrial Ca^2+^ uptake (Fig. 5, *E* and *F*).

These results demonstrate that the reintroduction of functional MCU nullifies differences in cytosolic Ca^2+^ upon activation of SOCE between WT cells and *Mcu*^−/−^ clones. Furthermore, expression of the MCU in *Mcu*^−/−^ cells fully rescues mitochondrial Ca^2+^ uptake. Both effects require a functional MCU ion channel pore.

### Reintroduction of MCU does not fully reverse the transcriptomic profile

**Figure 6:**
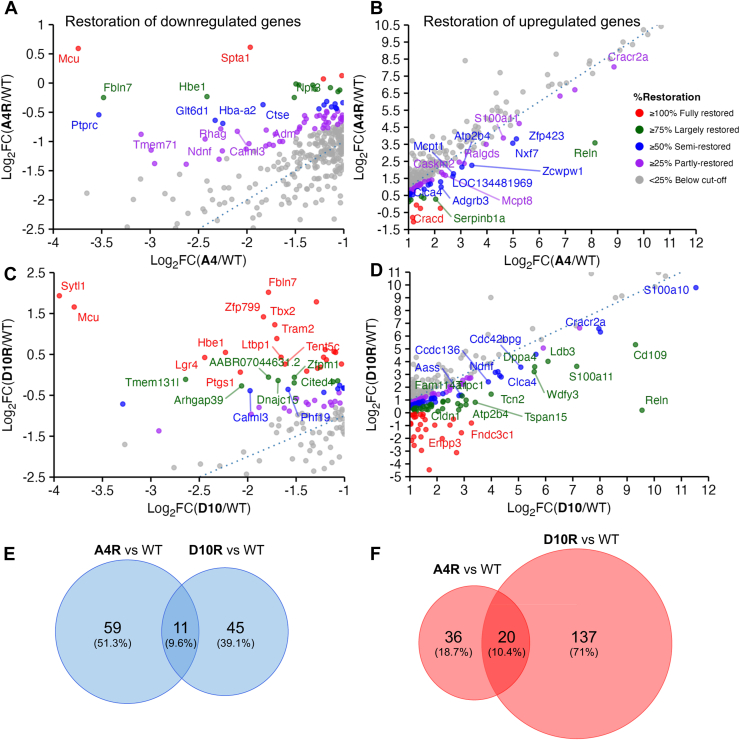
**Reintroduction of MCU partially restores transcriptomic changes in *Mcu*^−/−^ clones to varying extents.***A*–*D*, scatter plots show the restoration of downregulated (*A*, *C*) and upregulated (*B*, *D*) genes in *Mcu* knockout clones A4 (*A*, *B*) and D10 (*C*, *D*) after reintroduction of human MCU. The *x*-axis represents Log_2_ fold change (Log_2_FC) of DEGs in *Mcu*^−/−^ clones relative to WT, and the *y*-axis shows the corresponding Log_2_FC after MCU reexpression. Percentage restoration was calculated by first converting Log_2_FC values to linear fold change, then determining the fraction of the original A4 or D10 deviation from WT that was recovered upon MCU reexpression. A4R and D10R denote A4 and D10 clones reexpressing MCU, respectively. Data points are color-coded by restoration categories: fully restored (≥100%) in *red*, largely restored (≥75%) in *dark green*, semirestored (≥50%) in *blue*, partly restored (≥25%) in *purple*, and below cutoff (<25%) in *dark gray*. Selected genes with the highest degree of restoration in each category are labeled above the diagonal (for downregulated genes; *A*, *C*) or below the diagonal (for upregulated genes; *B*, *D*). *E*–*F*, Venn diagrams show the number of commonly and exclusively restored genes that were originally downregulated (*E*) and upregulated (*F*) in the *Mcu* KO clones A4 and D10, following the reexpression of human MCU. DEGs, differentially expressed genes; FC, fold change; MCU, mitochondrial Ca^2+^ uniporter.

We investigated whether expression of MCU was able to reverse the large changes in cell transcriptomics in the A4 and D10 *Mcu* KO clones so that they now better resembled WT cells. Reintroduction of human MCU restored expression of ∼20% of the genes downregulated in the A4 clone (70 out of 339), with varied degrees of recovery (≥25%) as shown in Figure 6, *A* and *E*. In the D10 clone, reintroduction of human MCU recovered expression in ∼40% of the downregulated genes (56 out of 144), with restoration levels of ≥25% (Fig. 6, *C* and *E*). Only 11 downregulated genes were commonly restored (Fig. 6*E*), including *Mcu* as expected. The fold-change heatmap of all these genes including *Mcu*, *Calml3*, and others is shown in Figure S7*A*.

In the transcriptome of the *Mcu* KO clone A4 re-expressing MCU, ∼20% of the genes (56 out of 265) that were upregulated in A4 relative to WT cells showed ≥25% restoration (Fig. 6, *B* and *F*). In contrast, ∼60% of the genes that were upregulated in clone D10 relative to WT (157 of 270) showed recovery in expression following reexpression of human MCU (Fig. 6, *D* and *F*). Among both *Mcu* KO clones reexpressing human MCU, 20 downregulated genes were commonly restored (Fig. 6*F*), including *Cracr2a*, *Reln*, *S100a11*, and *Atp2b4*, and their fold-change heatmap is shown in Figure S7*B*.

Restoration analysis indicated that clone D10 exhibited a greater degree of restoration of the transcriptomic changes. Volcano plots of WT, A4, and D10 with the respective cell lines reexpressing MCU revealed a larger number of differentially regulated genes in D10 (Fig. S8). Consistently, the MDS plot showed a greater Euclidean distance between D10 and D10 cells reexpressing human MCU (labeled D10R; Fig. S5*B*). The 31 genes (including *Mcu*) commonly restored in both clones following MCU reintroduction (Figs. 6, *E* and *F*, and S7) are likely directly affected by the loss of *Mcu*, rather than reflecting off-target effects of the CRISPR/Cas9 approach. Therefore, these genes represent components of the transcriptome that are dependent on the expression of MCU.

### PMCA activity is slowed in the absence of MCU

**Figure 7:**
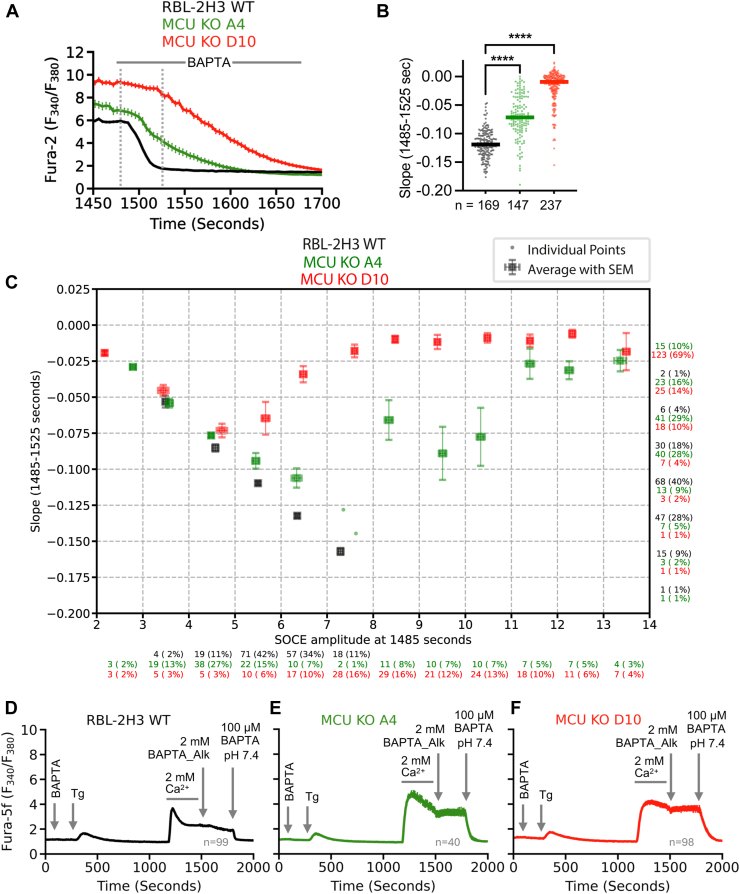
***Mcu* KO cells show delayed PMCA-mediated clearance of Ca^2+^.***A*, magnified view of the Ca^2+^ clearance phase of WT RBL-2H3 and *Mcu* KO clones A4 and D10 from Figure 3*E*. *B*, quantifications of rate of Ca^2+^ clearance phase, with medians presented as *solid lines*. *C*, scatter plots show the relationship between SOCE amplitude and the rate of cytosolic Ca^2+^ clearance in WT and *Mcu*^−/−^ clones. For each trace, SOCE amplitude was determined at the time point when Ca^2+^-free solution (plus 100 μM BAPTA) exchange was performed (at 1485 s), and the corresponding clearance slope was calculated by linear regression over the interval 1485 to 1525 s. Data points are grouped into amplitude bins (2–14) and slope bins (−0.20–0.025). Bins containing ≤2 traces are shown as individual points (*dots*), whereas bins with >2 traces are represented by mean values (*squares*) with standard error of mean (SEM) as error bars. Numbers of events per bin are indicated as counts and percentages under the *x*-axis (amplitude) and on the *right* side of the figure for *y*-axis (slope) in *black* for WT, and in *green* and *red* for A4 and D10, respectively. *D*–*F*, average traces (±SEM) from RBL-2H3 WT (*D*), *Mcu* KO A4 (*E*) and D10 (*F*) clones are compared, with all exchanges indicated. Ca^2+^-free solution (containing 100 μM BAPTA) at pH 11.0 is indicated as BAPTA_Alk. Briefly, 100 μM BAPTA denotes Ca^2+^-free solution containing 100 μM BAPTA at pH 7.4. MCU, mitochondrial Ca^2+^ uniporter; PMCA, plasma membrane Ca^2+^ ATPase; SOCE, store-operated Ca^2+^ entry.

The PMCAs are key regulators of cytosolic Ca^2+^ homeostasis, extruding excess Ca^2+^ from the cell following Ca^2+^ influx to maintain low basal cytosolic Ca^2+^ levels. Among these, PMCA4 (encoded by *Atp2b4*) is widely expressed and contributes significantly to Ca^2+^ extrusion in many cell types. Interestingly, RNA-seq analysis revealed that *Atp2b4* (PMCA4) was upregulated ∼8-fold in both *Mcu* knockout clones, A4 and D10, and its expression was partially restored upon MCU re-expression by approximately 50% in A4 and 90% in D10 (Fig. S7*B*). Upregulation of PMCA would be expected to increase calcium clearance in the *Mcu* KO cells. However, to our surprise, the clearance of Ca^2+^ appeared to be slower in both *Mcu* KO clones A4 and D10 (Figure 2, Figure 3, *C*–*E*). The slower clearance of Ca^2+^ may explain the larger cytosolic Ca^2+^ rise following SOCE in *Mcu* KO clones A4 and D10. We therefore carefully analyzed the Ca^2+^ clearance in WT and *Mcu*^−/−^ clones. A magnified view of the Ca^2+^ clearance phase from the experiments in Figure 3*E* is presented in Figure 7*A*. Quantifications of slopes of WT, A4, and D10 traces show a slower rate of Ca^2+^ clearance in the *Mcu* KOs (Fig. 7*B*).

Because the rate and extent of Ca^2+^ clearance is dictated by the size of the Ca^2+^ signal, we next compared Ca^2+^ clearance only for cytosolic Ca^2+^ signals of the same amplitude. After measuring Ca^2+^ clearance rate for each cell, we binned the cells into groups that showed the same cytosolic Ca^2+^ peak immediately prior to perfusion with Ca^2+^-free solution. Ca^2+^ clearance was substantially slowed in *Mcu*^−/−^ clones, when compared with WT cells (see 4–5, 5–6 and 6–7 amplitude bins). Our experimental design allowed us to identify which Ca^2+^ clearance pathway(s) was compromised following loss of MCU protein. In our studies, Ca^2+^ clearance by SERCA pumps was prevented by thapsigargin. The finding that ionomycin failed to release Ca^2+^ following exposure to FCCP demonstrates the absence of Ca^2+^ sequestration into acidic organelles, following a cytosolic Ca^2+^ rise induced by SOCE. RBL cells do not express functional Na^+^-Ca^2+^ exchangers in the plasma membrane (33). For WT cells, the remaining Ca^2+^ clearance pathways are therefore plasma membrane Ca^2+^ ATPase pumps and mitochondrial Ca^2+^ uptake, whereas for *Mcu*^−/−^ cells, it is only the plasma membrane pump. Selective inhibitors of the PMCA are lacking. However, PMCA-mediated exchange of cytosolic Ca^2+^ for extracellular H^+^ is inhibited under alkaline extracellular conditions (43, 44). Consistent with a predominant role for PMCA in Ca^2+^ clearance in *Mcu* KO cells, application of alkaline Ca^2+^-free buffer (pH 11.0) 5 min after the Ca^2+^ entry phase, completely blocked the clearance of Ca^2+^ in both *Mcu* KO clones A4 and D10 (Fig. 7, *E* and *F*). Switching back to Ca^2+^-free buffer (pH 7.4) cleared cytosolic Ca^2+^ from both the clones.

In WT cells, exposure to alkaline Ca^2+^-free solution also substantially delayed the recovery of cytosolic Ca^2+^, following a Ca^2+^ rise through store-operated Ca^2+^ channels (Fig. 7*D*). Therefore, in WT and *Mcu*^−/−^ cells, Ca^2+^ clearance is mainly *via* the PMCA pump. However, while impaired PMCA activity could be attributed to reduced ATP levels in *Mcu* KO cells, our measurements showed bulk ATP was not reduced in either clone compared with WT cells (Fig. S9).

### The increased cytosolic Ca^2+^ signal in *Mcu*^−/−^ cells is not due to clonal expansion-associated heterogeneity

We next asked whether the increased Ca^2+^ levels following SOCE observed in both single-cell-derived *Mcu* KO clones (A4 and D10) relative to WT cells could arise from clonal expansion-associated heterogeneity. Therefore, we tested two additional *Mcu* KO clones, D6 and B8, sister clones of A4 and D10 generated using gRNA2 and gRNA1, respectively. These *Mcu* KO clones exhibited a similar pattern of Ca^2+^ signal following store depletion as observed in A4 and D10, (Figs. S10 and 2*A*). Resembling the A4 clone, Ca^2+^ signals evoked by thapsigargin in the D6 clone were similar to WT responses, with a subset showing elevated responses (Fig. S10*B*). In the B8 clone, Ca^2+^ signals following stimulation with thapsigargin were considerably larger than those in WT cells, as was the case with the D10 clone (Fig. S10*C*).

To further address the possibility that the observed Ca^2+^ phenotype may have resulted from clonal expansion, we generated polyclonal *Mcu* KO and control RBL-2H3 cell populations without single-cell cloning. This was achieved by transducing cells with CRISPR/Cas9 lentiviral constructs carrying either gRNA2 (used to generate clone A4), gRNA1 (used to generate clone D10), or a scrambled control guide RNA.

Both gRNA1- and gRNA2-transduced polyclonal populations exhibited complete loss of MCU protein and recapitulated the enhanced cytosolic Ca^2+^ signaling following SOCE, along with reduced ionomycin-releasable mitochondrial Ca^2+^ pools compared with the scrambled control (Fig. S11). Because these results broadly recapitulate the findings obtained with the single-cell-derived A4 and D10 clones, we can rule out the possibility that clonal heterogeneity contributed to the amplified Ca^2+^ phenotype observed in the *Mcu* KO clones.

We next asked whether *MCU* loss produces a similar effect in another cell type by examining SOCE in HeLa cells. At a higher extracellular Ca^2+^ concentration (10 mM), *MCU* KO HeLa cells exhibited increased cytosolic Ca^2+^ levels following SOCE compared with WT cells, whereas very little difference was observed either in 0.5 mM or 2 mM Ca^2+^ (Fig. S12).

## Discussion

A substantial body of evidence has established that mitochondria regulate CRAC channels, and that knockdown of the MCU reduces SOCE and the subsequent cytosolic Ca^2+^ increase (24, 25, 26, 27, 28, 29, 30, 31, 32, 33, 34, 35, 36, 37). Recent work using CRISPR/Cas9-based deletion of the *Mcu* gene has yielded different results in that the cytosolic Ca^2+^ rise following SOCE increased substantially in *Mcu*^−/−^ cells (38, 39), due to an apparent increase in CRAC channel activity (39). Our data provide new insights into this issue. We found that the Ca^2+^ rise in *Mcu*^−/−^ cells following SOCE is significantly larger than in WT controls. However, this is not due to an increase in Ca^2+^ channel activity. Instead, the larger Ca^2+^ rise results from slower cytosolic Ca^2+^ removal when MCU is absent, with reduced Ca^2+^ clearance primarily reflecting altered plasma membrane Ca^2+^ ATPase pump-mediated extrusion.

One recent study included the RBL cell line to examine the impact of CRISPR/Cas9-mediated *Mcu* gene deletion on SOCE (38). We compared the gRNA used by Yoast *et al.* (38) with those used in our study. Interestingly, gRNA1 used to generate clone D10 showed ∼90% overlap with the gRNA reported in their study, targeting the region encoding residues 100 to 107 of MCU. CRISPOR (https://crispor.gi.ucsc.edu/) predicted that the gRNA used by Yoast *et al.* could have up to 30 potential off-target sites (1 with two mismatches, 5 with three mismatches, and 24 with four mismatches; only sites with up to four mismatches assessed), whereas our gRNA1 was predicted to have substantially fewer off-targets (5 sites, all with four mismatches). Without transcriptomic data, it therefore cannot be excluded that alterations in SOCE regulators contributed to the apparent increase in cytosolic Ca^2+^ following SOCE.

Our RNA-seq analysis directly addresses this possibility, revealing substantial transcriptomic remodeling following *Mcu* deletion. By identifying more than 200 DEGs shared between our two independent KO clones, and observing that ∼15% of these showed significant restoration toward WT levels in both clones upon MCU re-expression, we established a potential signature of MCU-dependent regulation. This dual-validation strategy allowed us to navigate the complexity of clonal variation and potential CRISPR/Cas9-associated alterations, effectively filtering out clone-specific artifacts. This narrowed our focus to candidates, such as the dramatic upregulation of *Cracr2a* and *Atp2b4* (PMCA4) that likely represent a direct consequence of disrupted mitochondrial Ca^2+^ handling rather than random genomic alterations. Importantly, we observed a similar enhancement of cytosolic Ca^2+^ levels following SOCE in additional *Mcu* KO clones and in polyclonal *Mcu* KO RBL-2H3 populations generated by lentiviral CRISPR/Cas9 delivery, indicating that the Ca^2+^ phenotype is highly unlikely to arise from clone-specific heterogeneity.

A larger cytosolic Ca^2+^ rise following Ca^2+^ entry through store-operated CRAC channels in *Mcu*^−/−^ cells could reflect increased channel activity, reduced cytosolic Ca^2+^ clearance, or a combination of both. Several findings argue against enhanced CRAC channel activity following deletion of the *Mcu* gene. First, Ba^2+^ readily permeates CRAC channels, does not evoke Ca^2+^-dependent inactivation of the channels, and is not transported out of the cytosol by Ca^2+^ ATPase pumps (42), making it a useful indicator of channel activity. Ba^2+^ flux through the channels was reduced, not increased, in *Mcu*^−/−^ clones compared with WT cells. Second, in 0.5 mM external Ca^2+^, the increase in cytosolic Ca^2+^ following SOCE was similar between WT and *Mcu*^−/−^ A4 cells. If CRAC channel activity had increased following *Mcu* deletion, A4 cells should have shown a larger cytosolic Ca^2+^ rise. D10 cells did show a larger Ca^2+^ rise, but the discrepancy between two cell lines lacking MCU suggests this difference is not entirely related to MCU loss. Third, *Orai1* expression was reduced in the A4 clone compared with WT cells, yet these cells showed larger cytosolic Ca^2+^ signals following SOCE, especially in the presence of 10 mM extracellular Ca^2+^. Fourth, both A4 and D10 *Mcu*^−/−^ clones exhibited larger increases in cytosolic Ca^2+^ following SOCE in the presence of 2 mM and particularly 10 mM extracellular Ca^2+^. By contrast, WT cells showed only a small increase when extracellular Ca^2+^ was raised. The pattern in A4 and D10 clones does not fit with the pore properties of the native CRAC channel. The K_D_ for Ca^2+^ permeation is ∼0.7 mM, and I_CRAC_ in 2 mM Ca^2+^ is only slightly smaller than in 10 mM (41). The different sizes of the cytosolic Ca^2+^ peaks following SOCE between *Mcu*^−/−^ clones and WT cells are therefore difficult to reconcile with the properties of the CRAC channel pore. Together, these observations argue against increased channel activity as the explanation for the larger Ca^2+^ signals.

Our RNA-seq data showed that *Cracr2a* was strongly upregulated (>450-fold in A4 and >250-fold in D10 *Mcu* KO clones) following *Mcu* deletion compared with WT cells. The Ca^2+^-binding protein CRACR2A forms a ternary complex with STIM1 and ORAI1, stabilizing STIM1-ORAI1 clusters (45). The complex dissociates when cytosolic Ca^2+^ increases. In *Mcu*^−/−^ cells, upregulation of CRACR2A could potentially stabilize STIM1-ORAI1 channel complexes, even when local Ca^2+^ near the channels is high, as occurs in 10 mM extracellular Ca^2+^. However, such a mechanism cannot explain the reduction in Ba^2+^ entry observed in KO clones, because Ba^2+^ is unlikely to substitute for Ca^2+^ in dissociating the CRACR2A-STIM1-ORAI1 complexes, and would therefore be expected to stabilize them. Regardless, *Mcu* deletion could alter the cytosolic Ca^2+^ rise following SOCE indirectly, through upregulation of CRACR2A and other channel modulators. Re-expression of MCU restored the expression of *Cracr2a* by more than 40% in A4 and ∼70% in *Mcu* KO clone D10, supporting the idea that the upregulation of *Cracr2a* is a downstream consequence of *Mcu* loss.

If CRAC channel activity has not increased in *Mcu*^−/−^ cells, then the large cytosolic Ca^2+^ rise following SOCE must reflect slower Ca^2+^ removal from the cytosol. Our measurements of Ca^2+^ clearance show that cytosolic Ca^2+^ is removed twice as fast in WT cells than in A4 cells and at least three times faster than in D10 cells. These findings differ from those in an earlier study, which reported accelerated Ca^2+^ clearance in *Mcu*^−/−^ cells (38). We do not know why our results differ, but Ca^2+^ clearance is an integrated process, reflecting various removal mechanisms in different cellular compartments with different affinities for Ca^2+^ and operating at different rates. It is therefore important to compare Ca^2+^ clearance for cytosolic Ca^2+^ signals only of the same cytosolic Ca^2+^ amplitude. We therefore binned our Ca^2+^ clearance results by size of the cytosolic Ca^2+^ rise and only compared WT cells and *Mcu*^−/−^ clones only within the same amplitude range. In contrast, the earlier study did not compare clearance at the same cytosolic Ca^2+^ levels (38), which could contribute to the discrepancy between studies. In addition, we isolated Ca^2+^ clearance by rapid removal of extracellular Ca^2+^, whereas those authors inhibited CRAC channels with Gd^3+^ in the continuous presence of extracellular calcium (38). Ca^2+^ clearance in their experiments will therefore involve the relative kinetics of inhibition of both CRAC channels and PMCA by Gd^3+^.

Because alkaline pH, which impairs PMCA activity, substantially reduced clearance in WT cells as well as in *Mcu*^−/−^ clones, this pump is the major determinant of Ca^2+^ extrusion under our conditions. How can its activity be affected by deletion of the *Mcu*? One possibility is that mitochondrial metabolism and ATP production are reduced in *Mcu*^−/−^ cells. A fall in cytosolic ATP would reduce PMCA activity. However, RBL cells can maintain intracellular ATP levels even when the electron transport chain is blocked, provided glucose is present extracellularly (46). We also did not observe any significant change in the ATP levels in our *Mcu* KO clones compared to WT. Consistent with this, we saw no difference in cytosolic Ca^2+^ clearance between WT and *Mcu*^−/−^ cells when thapsigargin was applied in Ca^2+^-free solution. If ATP levels had been compromised, cytosolic Ca^2+^ should have recovered more slowly in *Mcu*^−/−^ cells.

Rather than a change in plasma membrane Ca^2+^ ATPase activity, an alternative possibility is that the pump saturates as cytosolic Ca^2+^ increases in *Mcu*^−/−^ cells, due to the loss of mitochondrial Ca^2+^ buffering. The pump is activated by calmodulin and has a K_M_ for cytosolic Ca^2+^ of ∼ 0.1 μM (47, 48), only slightly above resting cytosolic Ca^2+^. In WT cells, mitochondrial uptake rapidly buffers Ca^2+^ entering through store-operated CRAC channels; this prevents a steep rise in cytosolic Ca^2+^, and works in concert with PMCA pumps to return the cytosol to resting levels. However, in *Mcu*^−/−^ cells which lack mitochondrial Ca^2+^ buffering, cytosolic Ca^2+^ rapidly reaches a significantly higher level than in WT cells following SOCE, and this would result in PMCA pump saturation and therefore a slowing in Ca^2+^ clearance. Such a mechanism could explain why WT and *Mcu* KO A4 cells have similar Ca^2+^ clearance rates in the presence of 0.5 mM Ca^2+^, because the rise in cytosolic Ca^2+^ is modest in both conditions and therefore can be handled by the PMCA.

Previous studies using an siRNA-based strategy to knock down the MCU found cytosolic Ca^2+^ was reduced in size following SOCE (32, 33, 34, 35, 36, 37), in sharp contrast to results drawn from *Mcu*^−/−^ cells (38, 39). SiRNA-based knockdown is invariably incomplete, but a major advantage is the short duration of knockdown studies, which limits long-term cellular adaptation. By contrast, knockout approaches often suffer from genetic compensation, with related genes being upregulated to counter the loss of a specific gene, allowing for canalization. Consistent with this, we found an increase in expression of PMCA4 transcripts in both *Mcu*^−/−^ clones, which may represent an adaptive response to mitigate against cytosolic Ca^2+^ overload.

In summary, by combining functional Ca^2+^ imaging with a rigorous transcriptomic filter, our findings demonstrate that increased cytosolic Ca^2+^ levels in *Mcu*^−/−^ cells arise primarily from slower Ca^2+^ clearance rather than a result of increased CRAC channel activity, and that compensatory upregulation of regulators like PMCA is insufficient to restore normal Ca^2+^ homeostasis. This work helps resolve the long-standing discrepancy between acute and chronic MCU loss and highlights a set of candidate MCU-dependent genes that may contribute to the regulation of cellular Ca^2+^ signaling. Future studies focusing on the shared transcriptional changes identified here may help further clarify how mitochondrial Ca^2+^ uptake influences Ca^2+^-dependent signaling networks and gene regulation.

## Experimental procedures

### Cell culture

RBL-2H3 cells (ATCC, CRL-2256) were cultured in Dulbecco’s minimum essential medium (DMEM, #11965–092, Gibco) supplemented with 10% premium heat-inactivated fetal bovine serum (#A5670801, Gibco), 2 mM glutamine (#25030081, Gibco), 1% Pen-Strep (#P0781, Millipore Sigma), hereafter referred to as complete DMEM (cDMEM). HeLa cells were cultured in RPMI 1640 (Gibco) medium supplemented with 10% premium heat-inactivated fetal bovine serum. Cells were maintained in a humidified 95% air, 5% CO_2_ incubator at 37 °C.

### CRISPR/Cas9-mediated knock out of *Mcu* in RBL-2H3 cells

Two different guide (g)RNAs targeting rat *Mcu* (Ensembl Gene ID: *ENSRNOG00000045920*; chromosomal location chr20 29,038,480—29,199,224 [-]; genome: Rnor_6.0 (rn6, *Rattus norvegicus*)) were designed using the Benchling CRISPR design webtool (https://benchling.com/crispr). The gRNAs were selected to target exon 3 and exon 4 of the rat *Mcu* transcript *ENSRNOT00000071477* (UniProt ID: M0RDI5), based on the best available ontarget (score 0–100, higher = better cutting efficiency) and offtarget (score 0–100, higher = lower risk of unintended cleavage) scores. The gRNA1 (5′-GCC TAT CTC GGA CTC CGT CG-3’; ontarget score: 62.8, offtarget score: 49.5, PAM: GGG) targeted a region encoding residues 101 to 108 (KPISDSVG) in exon 3, while gRNA2 (5′-GAG CAG GAG GTC TAT CCC CG-3’; on-target score: 74.5, offtarget score: 47.0, PAM: TGG) targeted a region encoding the residues 138 to 144 (TGIDLLL) in exon 4. Both gRNAs also targeted the alternative transcript *ENSRNOT00000090771* (UniProt ID: A0A0G2K059).

Rat *Mcu* gRNA1 oligonucleotides (forward: 5′-CAC CGC CTA TCT CGG ACT CCG TCG-3′ and reverse: 5′-AAA CCG ACG GAG TCC GAG ATA GGC-3′) and gRNA2 oligonucleotides (forward: 5′-CAC CGA GCA GGA GGT CTA TCC CCG-3′ and reverse: 5′-AAA CCG GGG ATA GAC CTC CTG CTC-3′) were synthesized by Integrated DNA Technologies (IDT). These complementary gRNA oligonucleotide pairs having BbsI compatible overhangs were annealed by incubating for 5 min at 95 °C in a thermocycler and then ramping down to 25 °C at 5 °C/min. The resulting double-stranded DNA guide inserts for gRNA1 and gRNA2 were cloned into BbsI-HF-digested and column-purified linearized vectors: pSpCas9(BB)-2A-GFP (PX458) (Addgene plasmid #48138) and pU6-(BbsI)_CBh-Cas9-T2A-mCherry (Addgene plasmid #64324), respectively, using T4 DNA ligase (New England Biolabs). The sequences of the final plasmids were verified by sequencing using 5′-GGA GAA AGG CGG ACA GGT AT-3′ forward primer (Genewiz, Azenta Life Sciences).

RBL-2H3 cells were transfected with each of the plasmids using the Cell Line Nucleofector Kit T (#VCA-1002, Lonza). Cells transfected with gRNA1/Cas9 and gRNA2/Cas9 were sorted by fluorescence-activated cell sorting based on GFP and mCherry fluorescence, respectively, and single cells were seeded into individual wells of separate 96-well plates. The single cell-derived clones were expanded, and the knockout was validated by both quantitative real-time PCR and western blotting.

The polyclonal populations of *Mcu* KO RBL-2H3 cells were generated by lentiviral transduction with gRNA1 or gRNA2 cloned into the spCas9 pLentiCRISPR v2 construct (GenScript). A scrambled guide RNA 5′-GCT TAG TTA CGC GTG GAC GA-3′ with no predicted genomic target sites (≤2-bp mismatches) in human, mouse or rat (49) was also cloned into spcas9 pLentiCRISPR v2 vector (GenScript). For each construct, 1 × 10^6^cells were transduced at a multiplicity of infection of 5 by the Viral Vector Core Facility at NIEHS. After culturing for 2 to 3 days, non-transduced cells were eliminated by puromycin selection (1.5 μg/ml for 8 days).

### CRISPR/Cas9-mediated knock out of *MCU* in HeLa cells

Guide (g)RNA targeting human *MCU* (Ensembl Gene ID: *ENSG00000156026*; chromosomal location chr10 72,692,131—72,887,694 [+]; genome: GRCh38 (hg38, *Homo sapiens*)) was designed using the Benchling CRISPR design webtool. The gRNA was selected to target exon 3 of the human *MCU* transcript *ENST00000373053* (UniProt ID: Q8NE86), based on the best available on-target (score 0–100, higher = better cutting efficiency) and offtarget (score 0–100, higher = lower risk of unintended cleavage) scores. The gRNA (5′-GGT TAC CTG TGA TAT CTG TG-3’; on-target score: 77.2, offtarget score: 64.4, PAM: AGG) targeted a region encoding residues 82 to 88 (GLPVISV) in exon 3. The human *MCU* gRNA also targeted the alternative transcripts *ENST00000357157*, *ENST00000536019* and *ENST00000604152*)

Human *MCU* gRNA oligonucleotides (forward: 5′-CAC CGG TTA CCT GTG ATA TCT GTG-3′ and reverse: 5′-AAA CCA CAG ATA TCA CAG GTA ACC-3′) were synthesized by Integrated DNA Technologies (IDT). These complementary gRNA oligonucleotide pairs having BbsI compatible overhangs were annealed by incubating for 5 min at 95 °C in a thermocycler and then ramping down to 25 °C at 5 °C/min. The resulting double-stranded DNA guide insert for human *MCU* gRNA was cloned into BbsI-HF-digested and column-purified pU6-(BbsI)_CBh-Cas9-T2A-mCherry (Addgene plasmid #64324) linearized vector using T4 DNA ligase (New England Biolabs). The sequence of the final plasmid was verified by sequencing using 5′-GGA GAA AGG CGG ACA GGT AT-3′ forward primer (Genewiz, Azenta Life Sciences).

HeLa cells were transfected with each of the plasmids using Lipofectamine 2000 (Invitrogen). Cells transfected with human *MCU* gRNA/Cas9 were sorted by fluorescence-activated cell sorting based on the mCherry fluorescence, and single cells were seeded into individual wells of separate 96-well plates. The single cell-derived clone F6 was expanded, and the knockout was validated by western blotting.

### Quantitative real-time PCR

Total RNA was isolated from WT RBL-2H3 cells and *Mcu* KO clones A4 and D10 using the RNeasy Mini Kit (#74104, Qiagen). RNA concentration and purity were assessed spectrophotometrically at 260 nm using NanoDrop 2000 (Thermo Fisher Scientific). One microgram of RNA was reverse transcribed into cDNA using the iScript cDNA Synthesis Kit (#1708890, Bio-Rad). Quantitative real-time reverse transcriptase-PCR was performed on a QuantStudio 3 system (Applied Biosystems) with TaqMan Universal PCR Master Mix (#4304437, Applied Biosystems) and gene-specific TaqMan assays (Rn01433739_m1 for *Mcu*, Rn01506496_m1 for *Stim1*, Rn02397170_m1 for *Orai1*, and Rn00667869_m1 for *Actb*). Reactions were carried out in MicroAmp Optical 96-well plates (Applied Biosystems), and data were analyzed using QuantStudio Design & Analysis Software v1.5.1 (Applied Biosystems). Relative expression levels were determined by the comparative ΔΔCt method and normalized to β-actin (*Actb*).

### Western blotting

RBL-2H3 cells were seeded at a density of 8 × 10^5^cells per well in Falcon tissue culture-treated 6-well plates (#353046, Corning). After 16 to 18 h of culture, the medium was removed, and cells were washed once with phosphate-buffered saline (PBS, pH 7.4). Cells were lysed in 800 μl Pierce radio-immuno precipitation assay (RIPA) buffer (#89901, Thermo Fisher Scientific) freshly supplemented with 1x cOmplete EDTA-free protease inhibitor cocktail (#11873580001, Roche) and 1 mM phenylmethanesulfonyl fluoride (PMSF; #93482, Sigma-Aldrich). Protein concentrations were determined using the Pierce BCA Protein Assay Kit (#23228, Thermo Fisher Scientific). Samples were adjusted to 2 μg/μl in RIPA buffer, mixed with 6x Laemmli SDS sample buffer (#J61337-AD, Thermo Fisher Scientific) to a final 1x concentration. Equal amounts of protein (40 μg per lane) together with SeeBlue Plus2 prestained standards (#LC5925, Invitrogen) were separated on NuPAGE 4 to 12% Bis-Tris gels (1.5 mm, #NP0335BOX, Invitrogen) using NuPAGE Mops SDS running buffer (#NP0001, Invitrogen) in a Novex Mini-Cell system (Invitrogen) at 100 V. Proteins were transferred to Immuno-Blot polyvinylidene fluoride membranes (0.2 μm pore size, 7.0 × 8.5 cm; #162–0218, Bio-Rad) pre-activated in 100% methanol for 1 min. Transfers were performed in 2x NuPAGE transfer buffer (#NP0006, Invitrogen) supplemented with 10% methanol and 1:1000 NuPAGE sample reducing agent (#NP0009, Invitrogen), using a Trans-Blot SD Semi-Dry Transfer Cell (Bio-Rad) at 20 V for 1 h (PowerPAC 300, Bio-Rad).

Membranes were blocked for 1 h at room temperature in 5% non-fat dry milk prepared in PBST (PBS containing 0.1% Tween-20), followed by overnight incubation at 4 °C with the following primary antibodies: rabbit monoclonal anti-MCU (1:1000; #14997, Cell Signaling), rabbit polyclonal anti-MCU (1:500; residues 47–152, #HPA016480, Millipore Sigma), rabbit polyclonal anti-DYKDDDDK (FLAG) tag (1:500; #PA1-984B, Invitrogen), and rabbit monoclonal anti-vinculin (1:1000; #13901, Cell Signaling) as loading control. After 3 to 4 washes (10 min each) with PBST, membranes were incubated for 1 h at room temperature with peroxidase-conjugated goat anti-rabbit IgG secondary antibody (1:5000; #XR-9920, ProSci). Following another three PBST washes, and one wash with PBS, signals were detected using SuperSignal West Pico PLUS chemiluminescent substrate (#34580, Thermo Fisher Scientific) and imaged on a ChemiDoc MP Imaging System (Bio-Rad) with Image Lab Touch Software v2.4.0.03 (Bio-Rad).

### Lentiviral transduction of RBL-2H3 cells with human *MCU*

Human *MCU*-Flag (Addgene plasmid #50054) was subcloned into the pCDH-EF1-MCS-T2A-copGFP lentiviral vector, and RBL-2H3 WT as well as *Mcu* KO A4 and D10 cells (1 × 10^6^cells) were transduced at a multiplicity of infection of 5 by the Viral Vector Core Facility at NIEHS. The D261Q and E264Q mutations in *MCU* within the pCDH-EF1-MCS-T2A-copGFP lentiviral vector were generated by GenScript, and both WT and *Mcu* KO cells were transduced with this construct under the same conditions.

### Single-cell calcium (and barium) imaging

RBL-2H3 WT cells, *Mcu* KO clones and their corresponding MCU-overexpressing rescue clones were seeded onto sterile round glass coverslips (30 mm diameter, 1.5 mm thickness; #30–1313–0319, Bioptechs) placed in Falcon tissue culture treated 6-well plates (#353046, Corning) at densities of 8 × 10^5^ or 3 × 10^5^cells per well, and cultured in cDMEM for an additional 24 or 48 h depending on whether the cells were to be analyzed the following day or 2 days post seeding. Fura-5F AM (#6616, Setareh Biotech, LLC) or Fura-2 AM (#F1221, Invitrogen) was dissolved in dimethyl sulfoxide (DMSO) to prepare 2 mM stock solutions. For calcium imaging experiments, coverslips with cultured cells were mounted in a Teflon chamber and incubated with 2 μM Fura-5F AM or 2 μM Fura-2 AM in DMEM (without Pen-Strep) for 30 min at room temperature in the dark.

The nominally Ca^2+^-free (NCF) solution consisted of a Hepes-buffered salt solution (HBSS) containing 145 mM NaCl (#S5150, Millipore Sigma), 2.8 mM KCl (#60142, Millipore Sigma), 2 mM MgCl_2_ (#AM9530G, Thermo Fisher Scientific), 10 mM Hepes (#16924, USB Corp) and 10 mM D-(+)-Glucose (#G8270, Millipore Sigma). The pH was adjusted to 7.4 with NaOH and the NCF solution was sterile-filtered. For use as the external bath solution, CaCl_2_ (#21115, Millipore Sigma) was added to the NCF solution at final concentrations of 0.5, 2, or 10 mM, as required.

Following loading with the Fura-based Ca^2+^ indicator, cells were washed twice with 1 ml of HBSS containing 2 mM CaCl_2_ and incubated for an additional 20 min at room temperature in the dark to allow for further de-esterification. Ratiometric calcium imaging data were acquired using InCyt Im2 v7.01 software (Intracellular Imaging Inc) on a Nikon Eclipse TS100 microscope. Prior to each experiment, regions of interest were defined to identify individual cells (50–100 cells per experiment), and separate regions of interest were designated for background subtraction. Fluorescence was recorded by alternately exciting the Fura indicator at 340 nm and 380 nm, with emission collected at 520 nm. Cytosolic Ca^2+^ levels are expressed as the F340/F380 fluorescence ratio. Data were acquired at a rate of 15 frames per minute. BAPTA (tetrasodium salt; #196418, Millipore Sigma) was dissolved in the NCF solution to prepare a 10 mM stock solution. A baseline was recorded for 2 min in HBSS containing 2 mM CaCl_2_, after which the solution was replaced with NCF solution containing 100 μM BAPTA, hereafter referred to as Ca^2+^-free (CF) solution, and data were acquired for another 3 min in CF solution. Thapsigargin (#1138, Tocris) was dissolved in DMSO to prepare a 2 mM stock solution. The CF solution was then replaced with freshly prepared CF solution containing 2 μM thapsigargin (Tg), and fluorescence was recorded for 15 min in Tg. Subsequently, the solution was exchanged with HBSS containing 2 mM CaCl_2_ and recorded for 5 min, followed by another exchange to CF solution and recording for an additional 10 min. Ionomycin (#407950, Millipore Sigma) was dissolved in DMSO to prepare a 10 mM stock solution. Finally, the bath solution was replaced with CF containing 10 μM ionomycin, and recordings were continued for another 5 min. To ensure complete solution exchange, each replacement was performed three times with 1 ml solution per exchange.

Fura-2 was selected for Ba^2+^ imaging because, despite its ∼3–4-fold lower affinity for Ba^2+^ (∼750 nM) compared to Ca^2+^ (∼200 nM), it retains sufficient sensitivity to detect intracellular Ba^2+^ entry. In contrast, Fura-5F, with a ∼2-fold lower Ca^2+^ affinity than Fura-2, binds Ba^2+^ even more weakly, rendering it unsuitable for monitoring Ba^2+^ influx. To measure Ba^2+^ entry following store-depletion, a 2 mM BaCl_2_ solution was prepared in CF HBSS and used for the add-back step.

### Calcium imaging on fluorometric imaging plate reader

RBL-2H3 WT and *Mcu* KO cells were seeded into black clear-flat-bottom 96-well tissue culture treated plates (#353219, Falcon/Corning) at a density of 4 × 10^4^cells per well in cDMEM. After 16 to 18 h of culture, the medium was replaced with 100 μl per well of cDMEM (without Pen-Strep) containing 3.5 μM Cal-520 AM calcium indicator (#21130, AAT Bioquest). A 50 μg vial of Cal-520 AM was first dissolved in 20 μl of DMSO and the entire volume was then added to 13 ml of cDMEM (without Pen-Strep) and mixed thoroughly to achieve a final Cal-520 AM concentration of 3.5 μM. Cells were incubated for 1 h in a humidified CO_2_ incubator at 37 °C. Following incubation, the medium was replaced with 100 μl per well of HBSS containing 2 mM CaCl_2_, and the cells were incubated at room temperature in the dark for 20 min to allow de-esterification. The cells were then washed once with 250 μl per well of CF HBSS and left in 100 μl of CF per well. Cells loaded with the Ca^2+^ indicator were excited using a 470 to 495 nm LED module of the Fluorometric Imaging Plate Reader (FLIPR Tetra; Molecular Devices), and the resulting fluorescence emission was collected through a 515 to 575 nm bandpass filter. The exposure time was set to 0.4 s, the camera gain to 90, and the excitation intensity to 90%. Data were acquired using ScreenWorks software version 4.2.0.10 (Molecular Devices).

After recording a 50 s baseline at an acquisition rate of 2 Hz, 50 μl of freshly prepared 6 μM Tg (3x stock) or DMSO (vehicle control) was administered and fluorescence was recorded for 1 min at 2 Hz, followed by an additional 9 min at 0.5 Hz. For Ca^2+^ add-back, 50 μl of 4x CaCl_2_ in HBSS was added to each well to achieve the desired final concentrations. Fluorescence was recorded at an initial rate of 2 Hz for 1 min followed by 0.5 Hz for the remainder of the recording period, up to a total of 15 min. Tg/DMSO and CaCl_2_ solutions were aspirated from polypropylene 96-well V-bottom microplates (#651201, Greiner Bio-One) containing 100 μl solution per well, ensuring sufficient liquid height (25 μl from the bottom) for reliable aspiration by the pipettor head. Fluorescence signals were analyzed using ScreenWorks 4.0.0.30 (Molecular Devices). Data were exported as response over baseline after background subtraction. The max-min values of the Ca^2+^ entry traces were also calculated using ScreenWorks.

For experiments involving the CRAC channel inhibitor BTP2, cells were incubated with 10 μM BTP2 or DMSO (vehicle control) during the 20 min de-esterification period following the 1 h Ca^2+^ indicator loading, by replacing the loading medium with CaCl_2_-containing HBSS supplemented with the respective treatment. The concentration of BTP2 was maintained throughout baseline recording and subsequent Tg trace acquisition.

### RNA-seq

#### RNA isolation and quality assessment

Total RNA was isolated in biological triplicates from RBL-2H3 WT and *Mcu* knockout clones (A4 and D10), and their respective MCU reexpression (rescue) counterparts (WTR, A4R, and D10R), using the RNeasy Mini Kit (Qiagen), including on-column DNase I digestion to remove genomic DNA contamination. RNA concentration was determined using Qubit fluorometry (Thermo Fisher Scientific), and RNA integrity was assessed using D1000 ScreenTape on the TapeStation 4200 with TapeStation Analysis Software 4.1 (Agilent Technologies).

#### Library preparation and sequencing

RNA-seq libraries were prepared and sequenced using a single-end 75 bp read format on the Illumina NextSeq 500 platform (Medium Output kit). The sequencing run yielded a total of 591,491,425 raw reads across 18 samples.

#### Quality control of raw reads

Quality control (QC) of raw sequencing reads was performed using FastQ Screen and FastQC (Babraham Bioinformatics, https://www.bioinformatics.babraham.ac.uk/projects/fastqc/) to verify high-quality sequences. QC metrics included assessments of per-base sequence quality, sequence duplication levels, GC content, k-mer content, sequence length distribution, adapter content, and per-sequence and per-tile quality scores. Quality scores were encoded using the Sanger/Phred+33 format.

#### Read alignment and gene quantification

The *R. norvegicus* (Norway rat) genome (accession GCF_015227675.2) was indexed using Spliced Transcripts Alignment to a Reference (STAR) v2.6.0c. Subsequently, the RNA-Seq reads from all samples were aligned to this reference genome, and gene counts were quantified directly during alignment using STAR’s --quantMode GeneCounts option.

#### Gene annotation

Following alignment and quantification of the 18 samples, the raw gene count matrices were imported into R 4.4.3 “Trophy Case”. Data handling and initial processing, including file reading, merging, and restructuring, were performed using readr, dplyr, purr, tibble, and tidyr packages from the Tidyverse v2.0.0 suite. Gene annotation was performed using the biomaRt v2.62.1 R package, querying the Ensembl BioMart database for *R. norvegicus* (rnorvegicus_gene_ensembl) to retrieve gene names and IDs. This annotation information was then merged with the raw counts. Genes in the raw count matrix that lacked a corresponding annotation were identified. Sample metadata were organized to define the following experimental groups: WT, A4, D10, WTR, A4R, and D10R.

#### Data filtering, normalization, and transformation

Raw gene count matrices were processed using the edgeR v4.4.2 package. After converting the count data into an edgeR DGEList object, genes with fewer than 200 counts in any sample were filtered out. The DGEList object was then normalized using the Trimmed Mean of M-values method. Normalized gene expression values were transformed to log_2_ Counts Per Million (log_2_CPM), with any nonfinite values removed. This log_2_CPM data were then merged with gene annotation information (ensembl_gene_id to external_gene_name) retrieved *via* the biomaRt package. Unannotated genes were also identified.

#### Unsupervised clustering

Following normalization and transformation, log_2_CPM expression data were used for unsupervised hierarchical clustering to assess sample relationships. The Euclidean distance was calculated between samples based on their log_2_CPM values. Hierarchical clustering was performed using the hclust function with the “average” linkage method. The resulting dendrogram was plotted with R’s base graphics, displaying sample relationships and relative distances.

#### MDS analysis

MDS plot was generated to assess the global relationships and similarity between samples. The normalized log_2_CPM expression data were used as input. The MDS plots were created using the plotMDS function from the limma v3.62.2 package (commonly used in conjunction with edgeR), visualizing the leading log-fold changes between samples across the most variable genes. Samples were colored according to their experimental groups (WT, A4, D10, WTR, A4R, and D10R) to visually inspect clustering and reproducibility.

#### Differential expression analysis and visualization

Differential expression analysis was performed using the edgeR package. A design matrix was constructed based on the experimental groups using an intercept-based model (*e.g.*, ∼group), and dispersions were estimated using the edgeR::estimateDisp function. A quasi-likelihood negative binomial generalized log-linear model was then fitted using glmQLFit(). Following this, a quasi-likelihood F-test (glmQLFTest) was then applied to identify DEGs for different pairwise comparisons, either by specifying coefficient indices or defining contrasts with contrast vectors. For each comparison, key metrics such as log-fold change, *p* value, and false discovery rate (FDR) were extracted using topTags() sorted by *p* value and gene IDs were subsequently converted to gene symbols by merging with the annotation data.

Volcano plots were generated using the ggplot2 v3.5.2 package for key comparisons to visualize differential gene expression. Genes were categorized for plotting based on a log_2_ Fold Change (FC) threshold of ±1 and an FDR threshold of 0.05 (figure legend for more details). Upregulated genes were defined as those with a log_2_FC ≥ 1 and FDR ≤ 0.05 in a specific comparison. Similarly, downregulated genes were defined by a log_2_FC ≤ −1 and FDR ≤ 0.05. The VennDiagram v 1.7.3 package was used to generate Venn diagrams for identifying common upregulated and downregulated genes in the two *Mcu* knockout clones or for the commonly restored genes after the reintroduction of *MCU*.

#### Pathway enrichment and visualization

Pathway analysis was performed using ingenuity pathway analysis (IPA) software v01.23.01. Canonical pathways meeting significance criteria (*p* value ≤ 0.05 and ∣z−score∣ ≥ 1.5) in at least one comparison (A4 *versus* WT or D10 *versus* WT) were selected for visualization. A bubble plot showing the enriched canonical pathways comparison, with pathways ordered by hierarchical clustering of their z-scores, was generated using R (ggplot2). Prior to clustering, missing z-scores were imputed as zero. Euclidean distance was used to compute pairwise dissimilarities between pathways, and clustering was performed using the complete linkage method. Pathways were filtered based on the following thresholds: –log10(*p* value) ≥ 1.3 (*i.e.*, *p* value ≤ 0.05) and absolute z-score ≥ 1.5 (*i.e.*, z-score ≥ 1.5 or ≤ −1.5).

#### Data availability

The raw data used for generating graphs presented in this manuscript are provided as source data files. All other data supporting the findings of this study are available from the corresponding author upon request.

## ATP quantification assay

RBL-2H3 WT and *Mcu* KO A4 and D10 cells were seeded into two black, clear, flat-bottom 96-well tissue culture-treated plates (#353219, Falcon/Corning) at a density of 5 × 10^3^cells per well in 100 μl cDMEM. One plate was used to measure ATP levels with the CellTiter-Glo 2.0 Assay (#G9241, Promega), following the manufacturer’s instructions, and luminescence was recorded using a LUMIstar OPTIMA microplate reader (BMG Labtech) with OPTIMA v2.20 software. The second plate was used to quantify cell number with the fluorescent nucleic-acid-binding dye-based CyQUANT Direct Cell Proliferation Assay (#C35011, Invitrogen), according to the manufacturer’s protocol. Fluorescence was measured on a Synergy 2 microplate reader (BioTek) with Gen5 v1.10.8 software using 485/20 nm excitation and 528/20 nm emission filter sets, bottom optics, and a sensitivity setting of 35. Cell counts obtained from the CyQUANT assay were used to normalize ATP levels.

## Mitochondrial membrane potential assay

Cells were seeded at a density of 300,000 per dish in 35 mm diameter, 1.5 mm thickness glass-bottom petri dishes (#P35G-1.5–14-C, MatTek). After culturing the cells in cDMEM for up to 48 h, the dishes were washed once with HBSS, and the cells were then loaded with 1 ml of 100 nM tetramethylrhodamine ethyl ester (TMRE) (#ab113852, Abcam) prepared in HBSS and incubated for 20 min in the dark at room temperature. The cells were washed twice, and a baseline was recorded in 500 μl HBSS for 200 s on the Zeiss LSM 880 confocal laser scanning microscope with an acquisition rate of 0.2 Hz. After recording baseline fluorescence, 500 μl of 5 μM FCCP (2x stock in HBSS) was added during imaging, and TMRE fluorescence was recorded for up to 10 min. The HBSS used in each step contained 2 mM CaCl_2_. Changes in mitochondrial membrane potential were quantified as (F – F_0_)/F_0_, where F_0_ represents the baseline fluorescence prior to FCCP addition. A decrease in TMRE fluorescence following FCCP treatment reflects the collapse of the mitochondrial membrane potential, and the extent of fluorescence loss corresponds to the degree of depolarization.

## Data availability

The data required for this manuscript are available either in the main article or in the supporting information file. Raw data for this manuscript will be provided on reasonable request.

## Supporting information

This article contains supporting information.

## Conflict of interest

The authors declare that they have no conflicts of interest with the contents of this article.
