# Supplementary material for: Deletion of mitochondrial calcium uniporter enhances calcium signals by slowing calcium clearance and triggers adaptive transcriptomic remodeling
Source: J Biol Chem. 2026 Apr 17;302(6):111473. doi: 10.1016/j.jbc.2026.111473 (PMC13197774; doi:10.1016/j.jbc.2026.111473)
Supplement: Supporting Information [file mmc1.pdf]

Supplementary Figure 1. Ca^{2+} entry in store-depleted *Mcu* KO RBL-2H3 cells is effectively blocked by CRAC channel blocker BTP2. Cytosolic Ca^{2+} recordings (average (± SEM)) taken from a Fluorometric Imaging Plate Reader (FLIPR) are compared for the conditions indicated. Cells were loaded with the Ca^{2+} indicator Cal520-AM (non-ratiometric) in WT RBL-2H3 (black), and *Mcu* KO clones A4 (green) and D10 (red). Cells were then pre-treated for 10 min with either DMSO or 10 µM CRAC channel blocker BTP2. The first Ca^{2+} transient reflects release of Ca^{2+} from the endoplasmic reticulum Ca^{2+} stores following the application of thapsigargin in Ca^{2+}-free buffer. The second Ca^{2+} peak occurs after adding back extracellular Ca^{2+} (2 mM). The Max-Min quantifications of the Ca^{2+} entry phase (n= 4 wells) are also shown and medians are presented as solid lines.

Supplementary Figure 2. SOCE enhances the ionomycin-releasable mitochondrial Ca^{2+} pool. (A, B) Ratiometric Ca^{2+} imaging traces (average ± SEM) of WT RBL-2H3 cells. All external solution exchanges are as indicated. BAPTA (100 µM) was applied in Ca^{2+}-free solution. Concentrations used were 2 µM Thapsigargin (Tg), 2 mM Ca^{2+}, 2 µM FCCP protonophore in BAPTA (B) and 10 µM Ionomycin (Iono). Ionomycin was applied in Ca^{2+}-free solution containing BAPTA. n = number of cells.

Supplementary Figure 3. Mitochondrial membrane potential was comparable between *Mcu* KO and WT RBL-2H3 cells. WT, A4, and D10 cells were loaded with TMRE (100 nM) to assess ΔΨm. Changes in TMRE fluorescence (before and after addition of 5 µM FCCP) were measured by confocal microscopy and quantified as $(F - F_0)/F_0$ are presented (n = 2 dishes).

Supplementary Figure 4. *Stim1* and *Orai1* expression in *Mcu* KO and WT RBL-2H3 cells. Quantitative Real-Time PCR analysis of expression levels of *Stim1* and *Orai1* in WT RBL-2H3 (gray), and *Mcu* KO A4 (green) and D10 (red) cells are compared. Medians are presented as bars and 95% confidence intervals as error bars (n=9; 3 biological replicates).

Supplementary Figure 5. Quality control plots of transcriptome replicates of RBL-2H3 WT and *Mcu* KO clones A4 and D10, and the rescue clones. (A) Hierarchical clustering dendrogram showing the relationships between biological replicates of RBL-2H3 WT, *Mcu* KO clones A4 and D10, and their respective rescue cell lines (WTR, A4R, and D10R). (B) Multidimensional scaling (MDS) plot of the same samples, where the proximity of points reflects transcriptional similarity. The color key for each cell line is shown.

Supplementary Figure 6. Low amounts of re-expressed MCU also rescue elevated cytosolic Ca^{2+} following SOCE in *$Mcu^{-/-}$* clones and refilling of mitochondrial pools. (A) Ratiometric Ca^{2+} imaging traces (average ± standard error of mean) are shown from single-cell experiments from WT (black) RBL-2H3 cells, *Mcu* KO clones A4 (green) and D10 (red) . (B) Ca^{2+} signals are compared between *Mcu* KO clone A4 expressing low levels of human MCU (light green) and *Mcu* KO clone D10 expressing low levels of human MCU (salmon). Thapsigargin (2 µM) was applied in extracellular solution containing 10 mM $CaCl_2$. (C) Quantifications show amplitude of the Ca^{2+} signal, measured from the last 30 seconds of the traces shown in A and B.

Solid lines represent the medians. (D-I) Average traces of Ca^{2+} signals from Ca^{2+} add-back assays in WT and *Mcu* KO clones A4 and D10 (D, G) and *Mcu* KO clone A4 expressing low levels of human MCU and *Mcu* KO clone D10 expressing low levels of human MCU (E, H). The buffer was exchanged from 2 mM $CaCl_2$ to Ca^{2+}-free solution containing 100 μM BAPTA (indicated by BAPTA in each trace), after which cells were exposed to 2 μM thapsigargin (Tg) in Ca^{2+}-free solution. Reintroduction of extracellular Ca^{2+} was performed with either 2 mM (D, E) or 10 mM (G, H) $CaCl_2$. Extracellularl Ca^{2+} was removed by switching to BAPTA-containing Ca^{2+}-free solution. 10 μM ionomycin (Iono) applied in Ca^{2+}-free buffer is indicated. Comparison of Max–Min values of Ca^{2+} entry phases shown in (F) for traces in panels D and E and in (I) for traces in panels G and H, were calculated by subtracting the minimum at 1150 s from the maximum at 1485 s (n = number of cells). Solid lines represent the medians. (J) Single-cell Ca^{2+} imaging traces from Ca^{2+} add-back assays in WT (black), *Mcu* KO clone D10 (red) and *Mcu* KO Clone D10 expressing low levels of human MCU (salmon). The solution was exchanged from 2 mM $CaCl_2$ to Ca^{2+}-free solution containing 100 μM BAPTA (indicated by BAPTA), after which cells were exposed to 2 μM thapsigargin (Tg) under Ca^{2+}-free conditions. 2 mM external $CaCl_2$ was added and Ca^{2+} measurements were continued for 70 more minutes after Ca^{2+} add-back. (K) Quantifications showing sustained levels of Ca^{2+}, averaged over the last 30 seconds of the traces shown in panel J. Solid lines represent the medians. Statistical significance was determined using either Student's t-test or the Mann-Whitney test, based on the outcome of the Shapiro–Wilk normality test. Significance levels are denoted as ns ($p > 0.05$), * ($p \leq 0.05$), ** ($p \leq 0.01$), *** ($p \leq 0.001$), and **** ($p \leq 0.0001$).

Supplementary Figure 7. Shared Restoration of Differentially Expressed Genes in *Mcu* KO Clones A4 and D10 after re-introduction of human MCU. Heat maps depict common genes with ≥25% restoration in both KO clones after MCU re-expression, relative to their downregulation (A) and upregulation (B) in the KO state. The fold change (Log_2FC) scale of genes shown on the right of the heatmaps (in each cell line type mentioned under the columns) is relative to the WT. *Mcu* KO clones A4 and D10 re-expressing MCU are referred to as A4R and D10R, respectively.

Supplementary Figure 8. Volcano plots compare differentially expressed genes in WT and *Mcu* KO Clones A4 and D10 of RBL-2H3 cells after re-introduction of MCU. Volcano plots of RBL-2H3 WT (A), *Mcu* KO clone A4 (B), and *Mcu* KO clone D10 (C) with the respective cell lines re-expressing MCU. Downregulated genes are shown in blue and upregulated in red. *Mcu* is indicated as green dot. Dark grey dots indicate genes having non-significant differences ($-log_{10}FDR < 1.3$) and light grey dots are the genes with $|log_2FC| < 1$.

Supplementary Figure 9. Relative ATP levels are compared between RBL-2H3 WT, A4 and D10 clones. Data are normalised to cell count and are presented as median bars with 95% confidence intervals as error bars (n=8 wells).

Supplementary Figure 10. RBL-2H3 *Mcu* KO clones D6 and B8, sister clones of A4 and D10, display elevated Ca^{2+} levels following entry after store depletion. (A) MCU immunoblot shows MCU expression in WT RBL-2H3, *Mcu* KO D6, and *Mcu* KO B8 cells. Vinculin served as a loading

control. Molecular weights are indicated in kilodaltons (kDa). (B, C) Ratiometric single-cell Ca^{2+} imaging traces (average ± standard error of mean) from RBL-2H3 WT (black), *Mcu* KO clone D6 (green) (B) and RBL-2H3 WT (black), *Mcu* KO clone B8 (red) are compared. Thapsigargin (2 µM) was applied in extracellular solution containing 2 mM $CaCl_2$. For each trace, the value at 240 s (pre-stimulation) was subtracted from the response at 1000 s (peak response); the resulting Max–Min values are quantified (n = number of cells) on the right of each Ca^{2+} trace. Solid lines represent the medians. Statistical significance was determined using either Student's t-test or the Mann-Whitney test, based on the outcome of the Shapiro–Wilk normality test. Significance levels are denoted as ns ($p > 0.05$), * ($p \leq 0.05$), ** ($p \leq 0.01$), *** ($p \leq 0.001$), and **** ($p \leq 0.0001$).

Supplementary Figure 11. Lentiviral CRISPR/Cas9-generated polyclonal *Mcu* knockout RBL-2H3 cells display enhanced cytosolic Ca^{2+} signaling following SOCE. (A) Western blot showing MCU expression in RBL-2H3 cells, after lentivirus transduction with a CRISPR/Cas9 construct carrying either scrambled control (Scr Ctr) gRNA, or gRNA2 (used to generate clone A4) labeled here as A4 gRNA Lenti, or gRNA1 (used to generate clone D10) labeled here as D10 gRNA Lenti. Vinculin served as a loading control. Molecular weights are indicated in kilodaltons (kDa). (B, C) Average (± SEM) Ca^{2+} imaging traces recorded on FLIPR using Cal520-AM Ca^{2+} indicator in Scr Ctr gRNA Lenti (black), A4 gRNA Lenti (green) and D10 gRNA Lenti (red) CRISPR/Cas9-transduced RBL-2H3 cells. Thapsigargin (2 µM) was applied in extracellular solution containing 2 mM $CaCl_2$ (B) or 10 mM $CaCl_2$ (C). (D, E) Average traces of Ca^{2+} signals from single-cell Ca^{2+} add-back assays in Scr Ctr gRNA Lenti, A4 gRNA Lenti or D10 gRNA Lenti transduced RBL-2H3 cells. The solution was exchanged from 2 mM $CaCl_2$ to Ca^{2+}-free solution containing 100 µM BAPTA (indicated by BAPTA in each trace), after which cells were exposed to 2 µM thapsigargin (Tg) under Ca^{2+}-free conditions. 2 mM external $CaCl_2$ was reintroduced at the indicated time point. Extracellular Ca^{2+} was removed by switching to BAPTA and a final application of 10 µM ionomycin (Iono) in Ca^{2+}-free buffer is indicated. Comparison of Max–Min values of Ca^{2+} entry phases are shown in (E) for traces in panel D, and were calculated by subtracting the minimum at 1150 s from the maximum at 1485 s (n = number of cells). Solid lines represent the medians. Statistical significance was determined using either Student's t-test or the Mann-Whitney test, based on the outcome of the Shapiro–Wilk normality test. Significance levels are denoted as ns ($p > 0.05$), * ($p \leq 0.05$), ** ($p \leq 0.01$), *** ($p \leq 0.001$), and **** ($p \leq 0.0001$).

Supplementary Figure 12. *MCU* KO in HeLa cells enhances the cytosolic Ca^{2+} signal following SOCE. (A) Western blot shows MCU expression in WT HeLa cells and *MCU* KO clone F6. Vinculin served as a loading control. Molecular weights are indicated in kilodaltons (kDa). (B) Average (± SEM) Ca^{2+} imaging traces recorded on FLIPR using Cal520-AM Ca^{2+} indicator in WT HeLa cells and *MCU* KO clone F6 are compared (n = number of wells). The cells were exposed to 2 µM thapsigargin (Tg) under Ca^{2+}-free conditions. 0.5 mM or 2 mM or 10 mM external $CaCl_2$ was applied at indicated time point(s). Max–Min values of Ca^{2+} entry phases were calculated by subtracting the minimum at 655 s from the maximum at 750 s. The respective Max–Min values (± Standard Deviation) for 0.5 mM, 2 mM, and 10 mM Ca^{2+} entry for WT vs *MCU* KO cells are 2.86 ± 0.14 vs 2.02 ± 0.23, 3.40 ± 0.08 vs 3.59 ± 0.32, and 3.87 ± 0.13 vs 4.82 ± 0.38.

Supplementary Table 1. Common Downregulated and Upregulated genes in *Mcu* KO clones A4 and D10.

Supplementary Figure 1

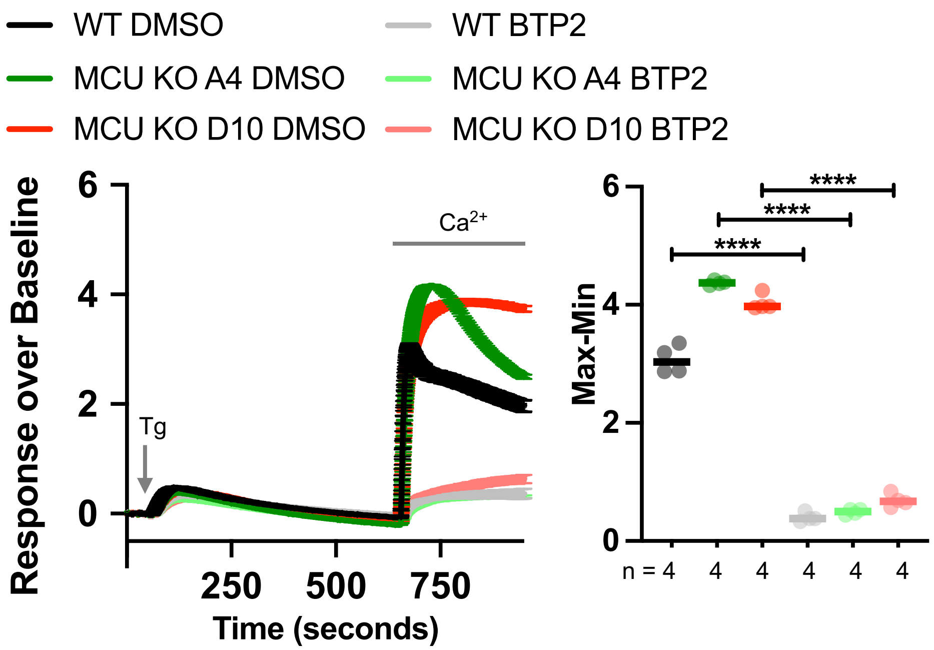

Supplementary Figure 2

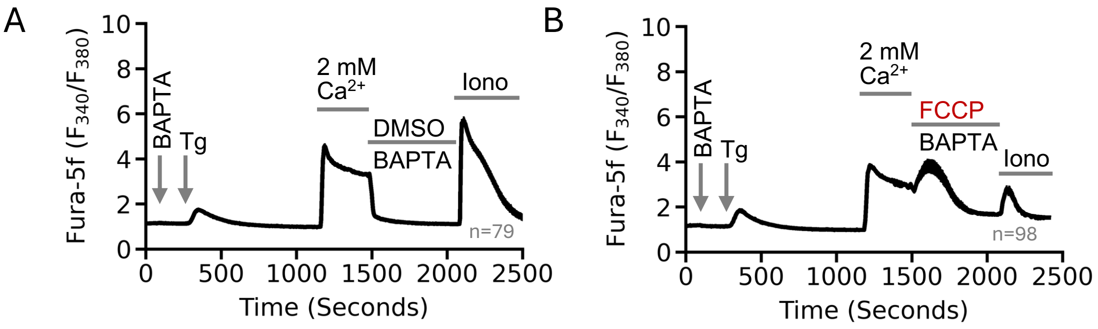

Supplementary Figure 3

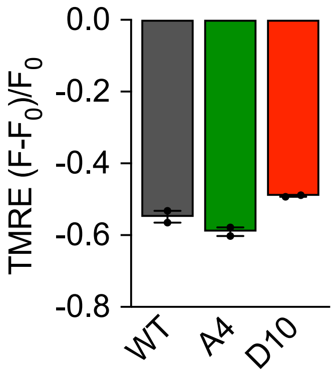

Supplementary Figure 4

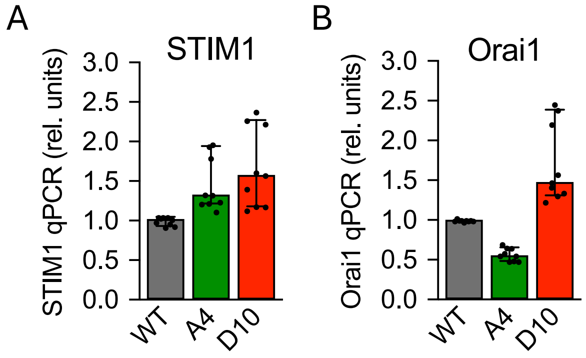

Supplementary Figure 5

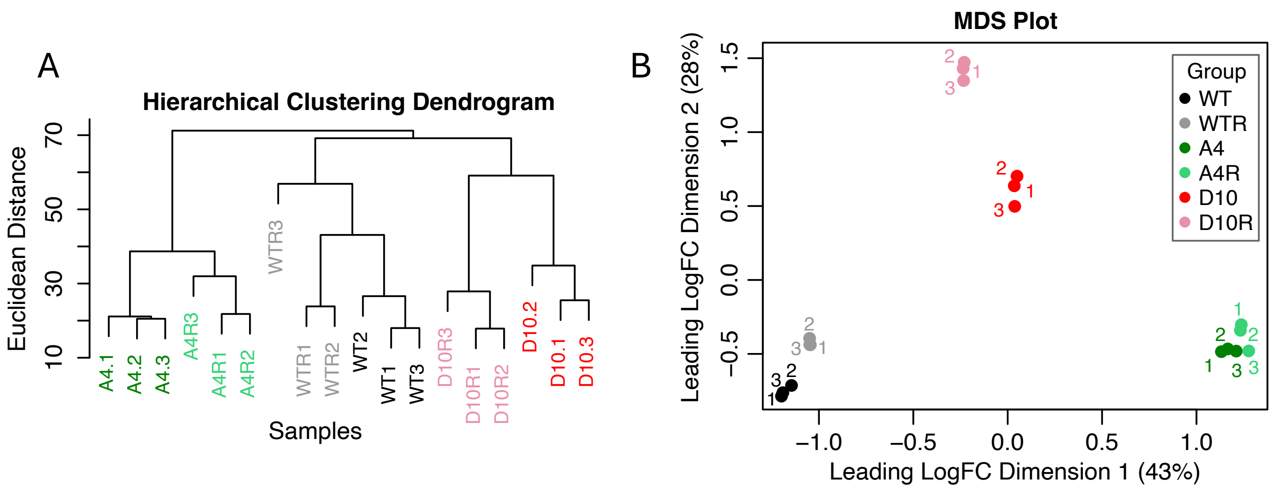

Supplementary Figure 6

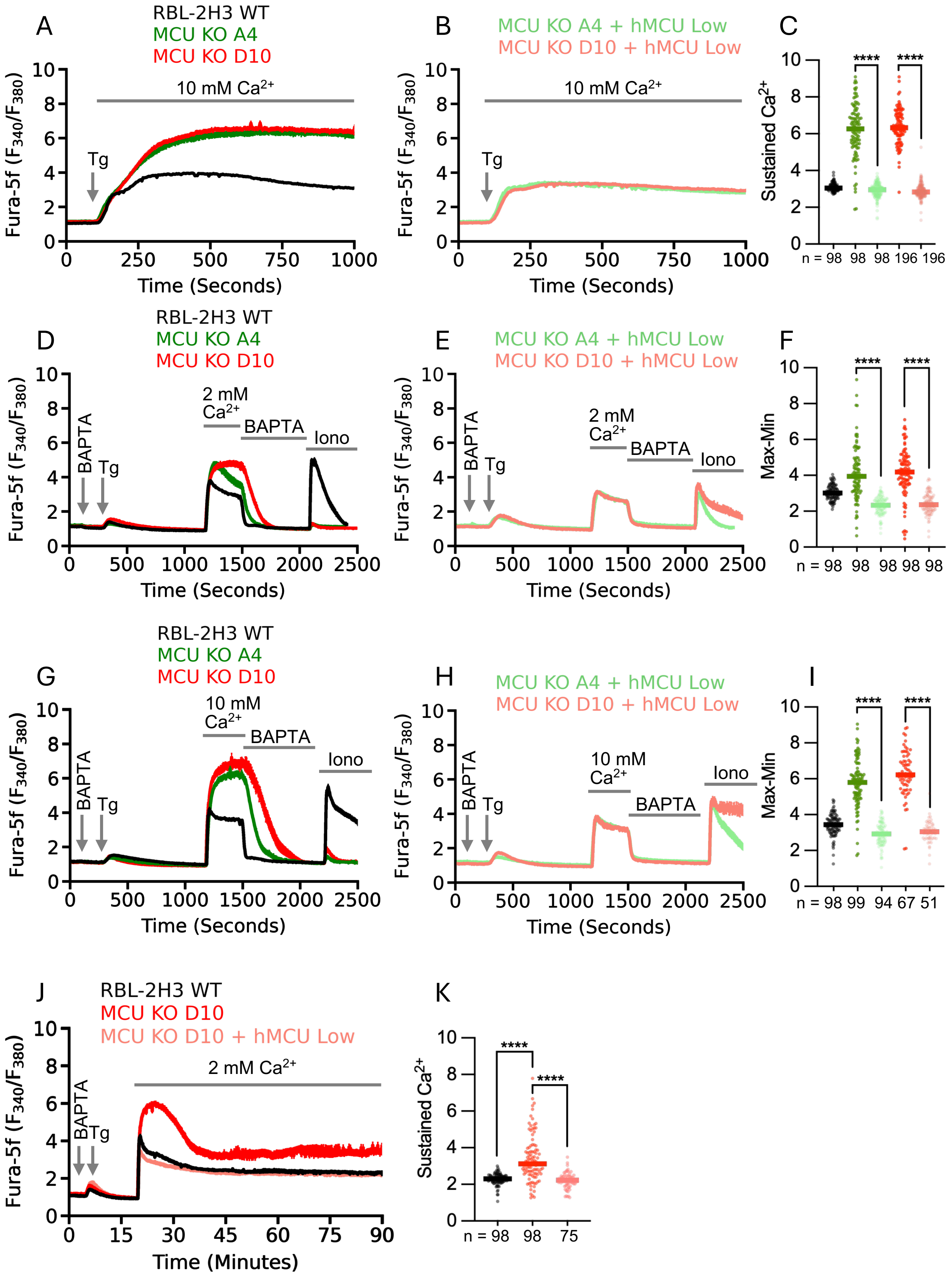

Supplementary Figure 7

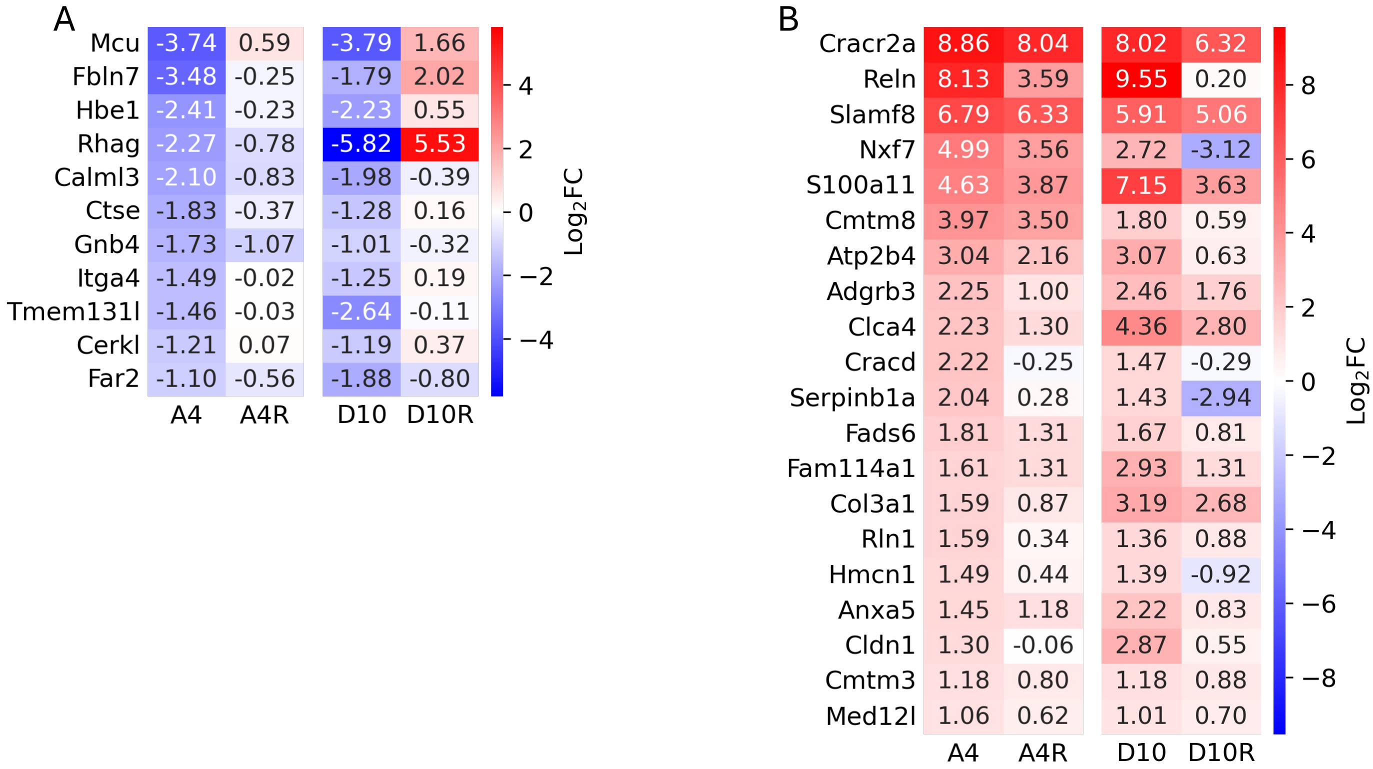

Supplementary Figure 8

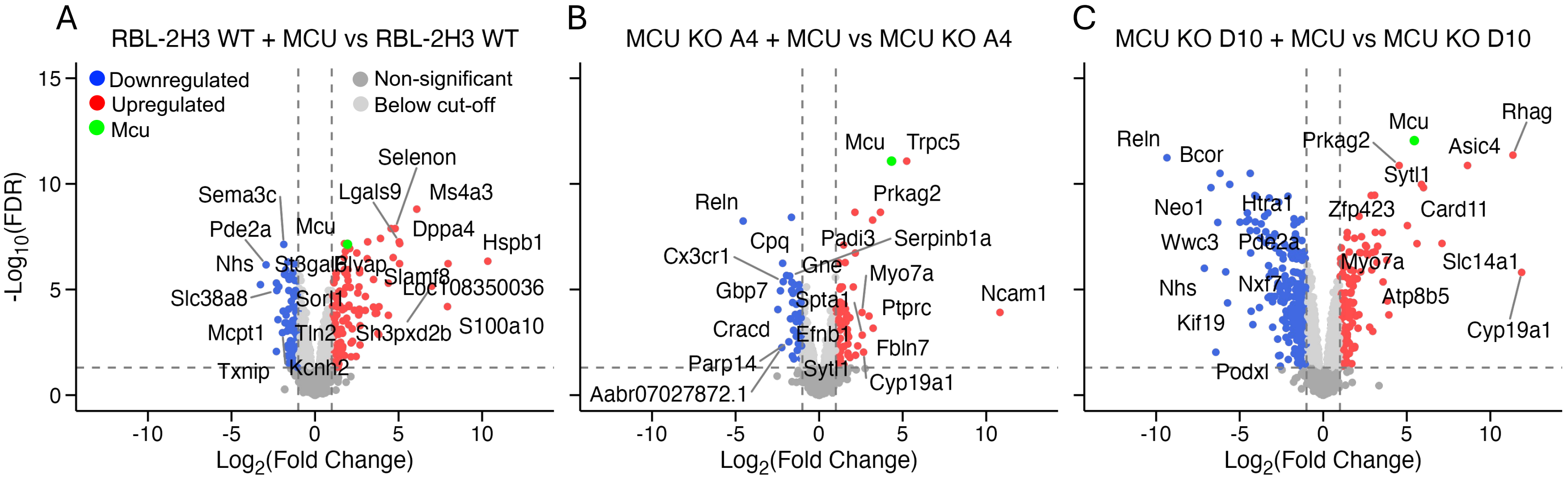

Supplementary Figure 9

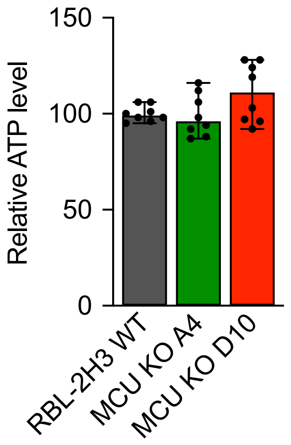

Supplementary Figure 10

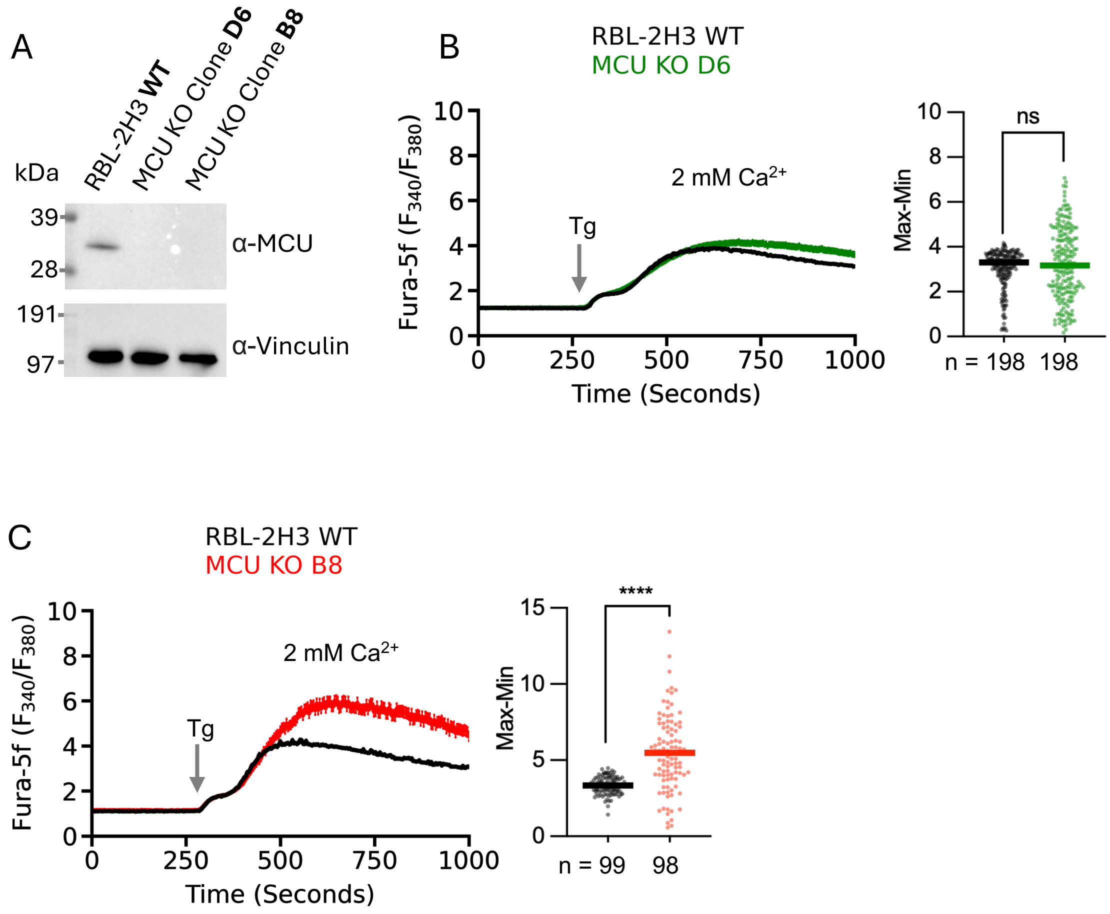

Supplementary Figure 11

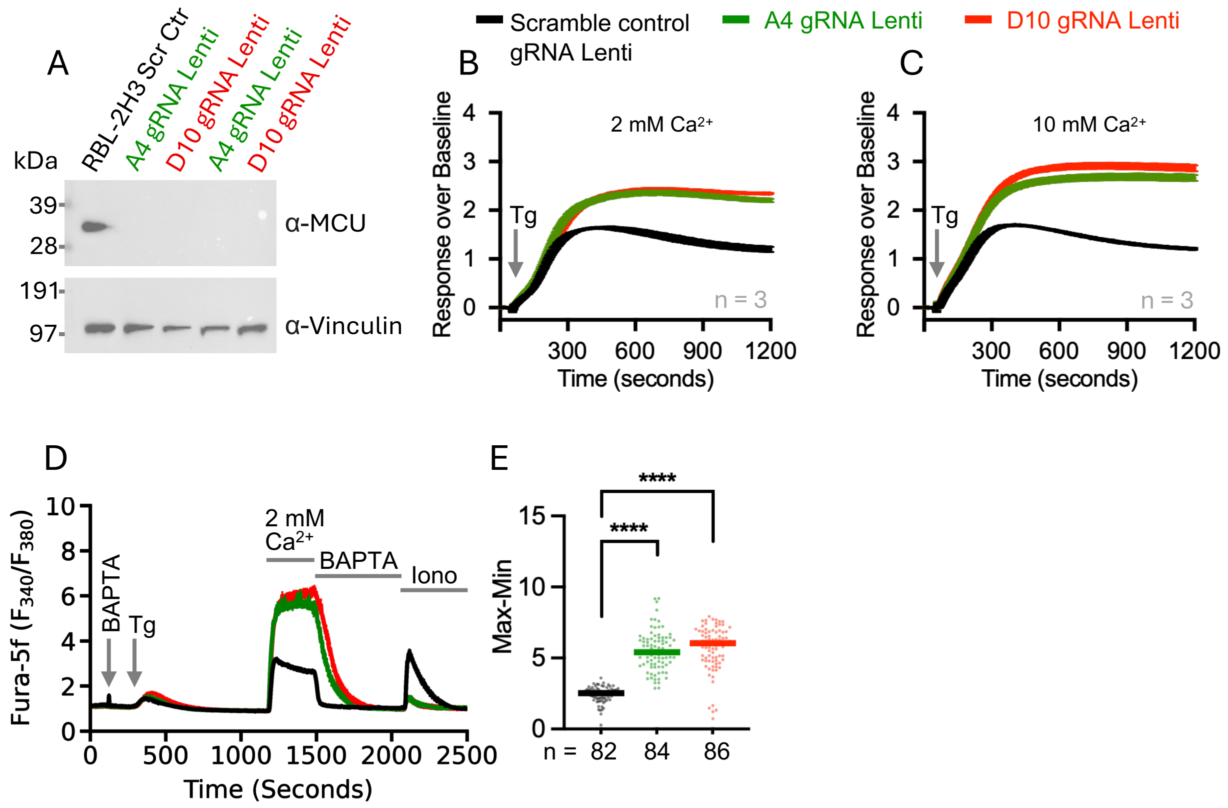

Supplementary Figure 12

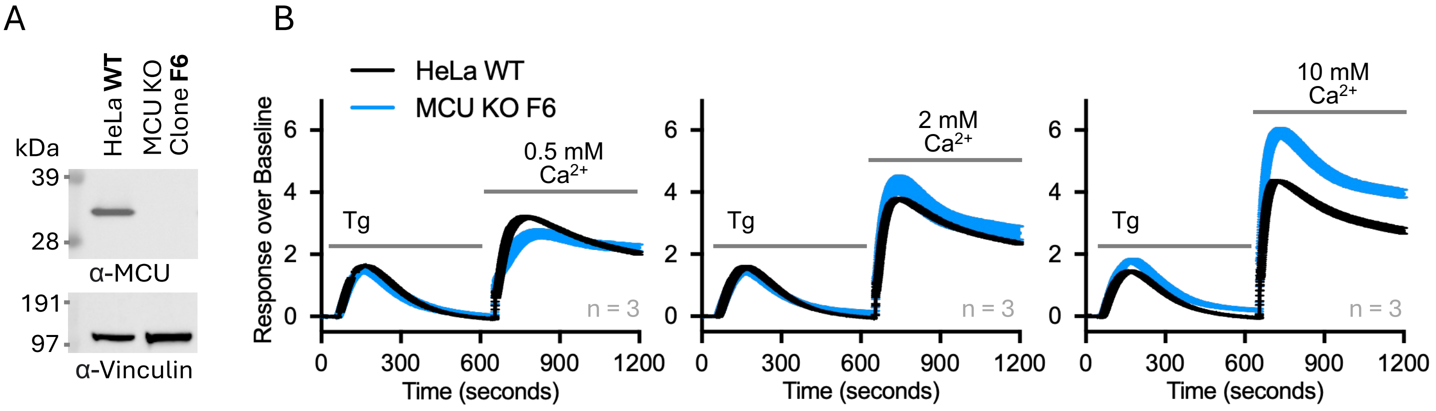

Supplementary Table 1: Common Downregulated and Upregulated genes in MCU KO clones A4 and D10

	GeneID	Gene	logFC.A4vWT	PValue.A4vWT	FDR.A4vWT	logFC.D10vWT	PValue.D10vWT	FDR.D10vWT
1	ENSRNOG00000020891	Kif9	-6.34	6.33E-16	1.41E-12	-5.71	4.28E-15	6.33E-12
2	ENSRNOG00000018874	Phf19	-3.76	5.10E-15	5.67E-12	-1.58	6.95E-10	9.08E-08
3	ENSRNOG00000045920	Mcu	-3.74	1.38E-14	1.22E-11	-3.79	1.70E-14	2.01E-11
4	ENSRNOG00000010668	Anxa6	-3.52	1.70E-14	1.37E-11	-3.55	1.81E-14	2.01E-11
5	ENSRNOG00000012495	Podxl	-9.31	2.43E-14	1.63E-11	-5.87	5.80E-13	3.68E-10
6	ENSRNOG00000023030	Cd96	-2.74	7.21E-13	2.29E-10	-2.49	3.51E-12	1.49E-09
7	ENSRNOG00000020533	Htra1	-3.17	8.34E-13	2.39E-10	-1.67	5.90E-09	5.35E-07
8	ENSRNOG00000029662	Wdfy4	-5.89	1.29E-12	3.28E-10	-1.34	4.47E-05	6.93E-04
9	ENSRNOG00000006963	Ctse	-1.83	2.07E-12	4.96E-10	-1.28	5.10E-10	7.19E-08
10	ENSRNOG00000008600	Sytl1	-5.70	2.63E-12	5.77E-10	-3.95	1.14E-10	2.11E-08
11	ENSRNOG00000012099	Tent2	-11.89	3.91E-12	8.09E-10	-11.89	1.39E-11	3.99E-09
12	ENSRNOG00000013484	Gsta1	-5.46	5.00E-12	9.87E-10	-1.50	1.07E-05	2.18E-04
13	ENSRNOG00000061582	Scamp1	-2.76	1.29E-11	1.91E-09	-1.07	4.11E-06	1.04E-04
14	ENSRNOG00000036960	Abcc9	-4.37	6.26E-11	7.85E-09	-5.85	3.43E-11	7.49E-09
15	ENSRNOG00000006649	Thrb	-4.08	5.31E-10	4.44E-08	-1.21	1.78E-04	2.07E-03
16	ENSRNOG00000021168	Anp32e	-1.03	9.93E-10	7.42E-08	-1.09	4.24E-10	6.29E-08
17	ENSRNOG00000019638	Lmna	-1.65	3.96E-09	2.26E-07	-1.70	2.62E-09	2.58E-07
18	ENSRNOG00000010450	Elovl7	-11.65	4.29E-09	2.38E-07	-11.65	1.01E-08	8.53E-07
19	ENSRNOG00000068198	C1h19orf84	-2.38	4.83E-09	2.60E-07	-1.13	4.24E-05	6.69E-04
20	ENSRNOG00000020202	Asrgl1	-2.24	1.43E-08	6.47E-07	-1.31	1.25E-05	2.49E-04
21	ENSRNOG00000024657	Mfsd4a	-2.72	2.53E-08	1.04E-06	-1.44	4.64E-05	7.17E-04
22	ENSRNOG00000046607	Cited4	-2.33	2.89E-08	1.16E-06	-1.52	6.29E-06	1.45E-04
23	ENSRNOG00000001296	P2rx7	-2.85	3.89E-08	1.44E-06	-3.27	1.76E-08	1.39E-06
24	ENSRNOG00000030212	Cerkl	-1.21	3.99E-08	1.46E-06	-1.19	6.18E-08	3.77E-06
25	ENSRNOG00000014678	Fzd5	-2.36	4.02E-08	1.46E-06	-1.67	3.16E-06	8.55E-05
26	ENSRNOG00000025503	Slc38a8	-2.36	4.91E-08	1.75E-06	-2.04	4.97E-07	2.10E-05
27	ENSRNOG00000000302	Sesn1	-1.14	5.42E-08	1.85E-06	-1.15	8.64E-08	4.89E-06
28	ENSRNOG00000000800	Man1a1	-1.02	6.23E-08	2.10E-06	-1.05	4.22E-08	2.84E-06
29	ENSRNOG00000000321	Cd24	-1.64	6.81E-08	2.23E-06	-2.18	2.92E-09	2.85E-07
30	ENSRNOG00000000655	Ptprc	-3.53	7.27E-08	2.34E-06	-1.79	7.71E-05	1.07E-03
31	ENSRNOG00000004441	Fcmr	-1.10	8.95E-08	2.81E-06	-1.98	2.06E-11	5.56E-09
32	ENSRNOG00000029658	Rnf213	-2.51	9.17E-08	2.83E-06	-5.00	7.44E-10	9.59E-08
33	ENSRNOG00000009675	Cdc42bpb	-2.28	9.23E-08	2.83E-06	-2.04	5.11E-07	2.11E-05
34	ENSRNOG00000020991	Ms4a6a	-1.06	1.18E-07	3.48E-06	-1.13	6.40E-08	3.84E-06
35	ENSRNOG00000008427	Necap2	-1.05	1.79E-07	4.78E-06	-1.25	1.82E-08	1.43E-06
36	ENSRNOG00000009837	Tmem131l	-1.46	2.16E-07	5.61E-06	-2.64	2.37E-10	3.90E-08
37	ENSRNOG00000031955	Calml3	-2.10	2.41E-07	6.10E-06	-1.98	1.24E-06	4.07E-05
38	ENSRNOG00000012061	Prkcb	-1.50	3.39E-07	8.08E-06	-1.01	4.16E-05	6.62E-04
39	ENSRNOG00000058422	Pirb	-1.39	4.69E-07	1.05E-05	-1.42	5.85E-07	2.31E-05
40	ENSRNOG00000055939	Zcchc18	-1.48	4.77E-07	1.07E-05	-1.32	3.87E-06	1.01E-04
41	ENSRNOG00000001094	Zfp316	-1.04	9.06E-07	1.80E-05	-1.07	8.53E-07	3.10E-05
42	ENSRNOG00000021185	Bola1	-1.33	9.15E-07	1.81E-05	-1.49	3.03E-07	1.40E-05
43	ENSRNOG00000017550	Fbln7	-3.48	1.55E-06	2.77E-05	-1.79	5.23E-04	4.70E-03
44	ENSRNOG00000019508	Wars2	-1.03	1.71E-06	2.99E-05	-1.39	7.29E-08	4.20E-06
45	ENSRNOG00000069699	Slc25a53	-1.35	2.23E-06	3.71E-05	-2.01	6.60E-08	3.89E-06
46	ENSRNOG00000023453	Lrba	-1.11	3.10E-06	4.95E-05	-1.25	7.39E-07	2.79E-05
47	ENSRNOG00000011070	Gnb4	-1.73	3.10E-06	4.95E-05	-1.01	8.64E-04	6.93E-03
48	ENSRNOG00000007422	Vav2	-1.26	3.35E-06	5.24E-05	-1.37	1.98E-06	5.88E-05
49	ENSRNOG00000020431	Matk	-1.12	3.71E-06	5.72E-05	-1.62	5.26E-08	3.39E-06
50	ENSRNOG00000064749	Sowahc	-1.45	7.18E-06	9.80E-05	-1.36	2.14E-05	3.86E-04
51	ENSRNOG00000063854	Ppa1	-1.02	8.90E-06	1.16E-04	-1.21	1.18E-06	4.02E-05
52	ENSRNOG00000043077	Zfpm1	-1.23	1.35E-05	1.61E-04	-1.52	1.24E-06	4.07E-05
53	ENSRNOG00000028350	Arsl	-1.58	3.58E-05	3.51E-04	-1.45	1.30E-04	1.64E-03
54	ENSRNOG00000050121	Rhag	-2.27	5.52E-05	5.00E-04	-5.82	1.48E-06	4.63E-05
55	ENSRNOG00000028534	Zfp518b	-1.12	7.56E-05	6.40E-04	-11.44	1.32E-10	2.35E-08
56	ENSRNOG00000004861	Itga4	-1.49	8.19E-05	6.87E-04	-1.25	4.73E-04	4.40E-03
57	ENSRNOG00000029286	Hbe1	-2.41	1.00E-04	8.05E-04	-2.23	4.53E-04	4.25E-03
58	ENSRNOG00000004150	Slc9a7	-1.19	1.36E-04	1.03E-03	-1.11	3.72E-04	3.66E-03
59	ENSRNOG00000062564	LOC120098765	-1.64	1.41E-04	1.06E-03	-1.07	5.18E-03	2.61E-02
60	ENSRNOG00000011321	Rftn1	-1.09	2.42E-04	1.65E-03	-1.15	2.78E-04	2.91E-03
61	ENSRNOG00000023312	Lonrf2	-1.04	4.65E-04	2.83E-03	-1.29	6.92E-05	9.84E-04
62	ENSRNOG00000038600	Dnaaf3	-1.19	7.32E-04	4.13E-03	-1.32	6.33E-04	5.44E-03
63	ENSRNOG00000001851	Far2	-1.10	1.27E-03	6.52E-03	-1.88	7.05E-06	1.56E-04
64	ENSRNOG00000019985	Asic4	-1.70	2.77E-03	1.24E-02	-4.37	4.40E-05	6.85E-04

	GeneID	Gene	logFC.A4vWT	PValue.A4vWT	FDR.A4vWT	logFC.D10vWT	PValue.D10vWT	FDR.D10vWT
65	ENSRNOG00000053449	Cracr2a	8.86	7.99E-18	7.10E-14	8.02	1.86E-16	1.65E-12
66	ENSRNOG00000059166	Ldb3	10.15	9.76E-16	1.73E-12	6.09	8.44E-10	1.07E-07
67	ENSRNOG00000015941	Fkbp10	8.71	2.48E-15	3.15E-12	4.28	2.46E-06	6.88E-05
68	ENSRNOG00000027936	Ccdc8	9.08	7.21E-15	7.12E-12	4.15	4.23E-04	4.03E-03
69	ENSRNOG00000027456	Cdc42bpg	7.91	2.37E-14	1.63E-11	5.09	1.29E-07	6.86E-06
70	ENSRNOG00000047781	Slc25a23	3.22	2.57E-14	1.63E-11	1.80	6.26E-10	8.43E-08
71	ENSRNOG00000023941	Fam178b	6.65	3.64E-14	2.16E-11	2.80	4.08E-03	2.20E-02
72	ENSRNOG00000004063	Sh3pxd2b	10.66	3.99E-14	2.22E-11	10.41	8.87E-14	7.89E-11
73	ENSRNOG00000025404	Dppa4	6.79	5.23E-14	2.74E-11	5.60	6.98E-12	2.39E-09
74	ENSRNOG00000070319	Zfp950l4	7.93	1.20E-13	5.44E-11	7.96	1.35E-13	1.09E-10
75	ENSRNOG00000004201	Rims2	8.04	1.22E-13	5.44E-11	8.81	4.25E-15	6.33E-12
76	ENSRNOG00000061121	Wdfy3	7.04	1.64E-13	6.95E-11	5.58	5.22E-10	7.25E-08
77	ENSRNOG00000025930	Chst13	4.90	1.99E-13	7.69E-11	1.42	5.97E-04	5.20E-03
78	ENSRNOG00000021441	Reln	8.13	2.99E-13	1.11E-10	9.55	7.16E-16	2.54E-12
79	ENSRNOG00000025324	Spire1	1.88	3.71E-13	1.32E-10	1.35	7.28E-11	1.47E-08
80	ENSRNOG00000008736	Slamf8	6.79	4.13E-13	1.41E-10	5.91	2.83E-11	6.61E-09
81	ENSRNOG00000023256	Nxf7	4.99	4.30E-13	1.42E-10	2.72	1.08E-06	3.75E-05
82	ENSRNOG00000034240	Bcor	3.22	7.78E-13	2.34E-10	1.71	1.15E-08	9.53E-07
83	ENSRNOG00000004137	Ubtd2	5.16	1.35E-12	3.33E-10	3.71	2.28E-09	2.36E-07
84	ENSRNOG00000009052	Igf2bp3	1.52	5.95E-12	1.13E-09	2.49	2.96E-15	6.33E-12
85	ENSRNOG00000053086	Selenop	6.36	6.29E-12	1.14E-09	7.25	4.02E-13	2.98E-10
86	ENSRNOG00000002451	Fndc3c1	3.87	6.74E-12	1.17E-09	3.27	1.12E-10	2.11E-08
87	ENSRNOG00000046421	LOC108350036	9.21	9.88E-12	1.54E-09	10.03	7.58E-13	4.49E-10
88	ENSRNOG00000001115	Slc29a4	2.48	1.24E-11	1.87E-09	2.39	2.54E-11	6.10E-09
89	ENSRNOG00000023993	Kif1a	3.02	1.80E-11	2.59E-09	1.16	5.20E-05	7.84E-04
90	ENSRNOG00000008133	Mfng	3.57	2.86E-11	3.85E-09	2.96	1.38E-09	1.55E-07
91	ENSRNOG00000020349	Rab3il1	3.51	6.96E-11	8.36E-09	1.17	2.10E-03	1.35E-02
92	ENSRNOG00000008941	Ets1	1.84	8.12E-11	9.63E-09	1.13	9.67E-08	5.34E-06
93	ENSRNOG00000014479	Cttnbp2nl	3.98	9.08E-11	1.02E-08	3.43	2.11E-09	2.20E-07
94	ENSRNOG00000009298	Fbxo44	2.78	1.28E-10	1.38E-08	2.37	2.54E-09	2.56E-07
95	ENSRNOG00000014909	Sh2d5	3.42	1.52E-10	1.57E-08	1.13	2.58E-03	1.57E-02
96	ENSRNOG00000008919	Arpp21	4.35	1.85E-10	1.87E-08	2.19	5.68E-04	5.01E-03
97	ENSRNOG00000042283	Zfp267	2.14	2.36E-10	2.33E-08	2.47	2.92E-11	6.66E-09
98	ENSRNOG00000019319	Fchsd2	3.03	2.45E-10	2.39E-08	2.03	3.19E-07	1.46E-05
99	ENSRNOG00000021380	Fads6	1.81	2.50E-10	2.41E-08	1.67	1.09E-09	1.31E-07
100	ENSRNOG00000021269	Chgb	4.42	3.34E-10	3.12E-08	5.64	1.91E-12	9.14E-10
101	ENSRNOG00000005747	Il27ra	4.11	3.46E-10	3.21E-08	3.25	7.01E-08	4.10E-06
102	ENSRNOG00000010625	Dnmt3b	9.99	3.50E-10	3.21E-08	7.68	1.40E-06	4.49E-05
103	ENSRNOG00000049277	Syne4	2.26	3.92E-10	3.52E-08	1.15	1.13E-05	2.28E-04
104	ENSRNOG00000003544	Tbx4	7.36	4.00E-10	3.52E-08	8.57	4.44E-12	1.79E-09
105	ENSRNOG00000015024	Mcoln3	6.32	4.38E-10	3.81E-08	3.87	1.32E-04	1.66E-03
106	ENSRNOG00000016581	Serpinb1a	2.04	4.49E-10	3.88E-08	1.43	7.32E-08	4.20E-06
107	ENSRNOG00000054902	Trpc1	3.51	4.82E-10	4.12E-08	3.07	6.51E-09	5.85E-07
108	ENSRNOG00000003031	Atp2b4	3.04	5.06E-10	4.28E-08	3.07	6.19E-10	8.43E-08
109	ENSRNOG00000016313	Ctnnbip1	2.42	5.38E-10	4.44E-08	2.00	1.62E-08	1.31E-06
110	ENSRNOG00000011201	Cmtm8	3.97	6.21E-10	5.02E-08	1.80	3.94E-04	3.81E-03
111	ENSRNOG00000028356	Chchd10	2.12	6.32E-10	5.06E-08	1.17	5.87E-06	1.38E-04
112	ENSRNOG00000070518	Gdf1	1.75	6.64E-10	5.27E-08	1.73	8.77E-10	1.10E-07
113	ENSRNOG00000046333	AABR07007032.1	3.00	9.38E-10	7.06E-08	3.27	2.86E-10	4.38E-08
114	ENSRNOG00000023226	S100a10	9.57	1.15E-09	8.54E-08	11.53	2.58E-12	1.15E-09
115	ENSRNOG00000010105	S100a11	4.63	1.30E-09	9.44E-08	7.15	3.77E-14	3.72E-11
116	ENSRNOG00000010219	Ralgds	3.13	1.36E-09	9.69E-08	1.24	1.32E-03	9.56E-03
117	ENSRNOG00000001653	St3gal6	2.05	1.50E-09	1.03E-07	1.16	7.44E-06	1.64E-04
118	ENSRNOG00000023546	Hspb1	10.16	1.51E-09	1.03E-07	10.16	2.09E-09	2.20E-07
119	ENSRNOG00000012045	Adgrb3	2.25	1.51E-09	1.03E-07	2.46	4.62E-10	6.74E-08
120	ENSRNOG00000006353	Large2	3.68	1.79E-09	1.16E-07	2.21	1.90E-05	3.51E-04
121	ENSRNOG00000010737	Mbnl2	4.39	2.28E-09	1.43E-07	3.05	1.23E-06	4.07E-05
122	ENSRNOG00000046850	Thpo	2.21	2.39E-09	1.47E-07	2.87	3.46E-11	7.49E-09
123	ENSRNOG00000012681	Lgals9	4.20	2.44E-09	1.50E-07	5.52	5.65E-12	2.09E-09
124	ENSRNOG00000017462	Afg2a	1.21	2.61E-09	1.55E-07	1.04	2.75E-08	2.02E-06
125	ENSRNOG00000012794	Grhpr	1.95	2.79E-09	1.64E-07	1.89	5.75E-09	5.27E-07
126	ENSRNOG00000004873	Prkch	3.00	4.14E-09	2.33E-07	1.08	5.67E-03	2.79E-02
127	ENSRNOG00000007100	Ccdc136	3.53	4.23E-09	2.37E-07	3.89	1.09E-09	1.31E-07
128	ENSRNOG00000015157	Smtnl2	2.01	5.61E-09	2.97E-07	1.61	1.82E-07	8.97E-06
129	ENSRNOG00000065530	LOC120094625	1.64	7.18E-09	3.65E-07	1.61	1.08E-08	9.07E-07

	GeneID	Gene	logFC.A4vWT	PValue.A4vWT	FDR.A4vWT	logFC.D10vWT	PValue.D10vWT	FDR.D10vWT
130	ENSRNOG00000002001	Itsn1	1.57	1.05E-08	5.08E-07	2.51	8.60E-12	2.83E-09
131	ENSRNOG00000004310	Caskin2	2.33	1.13E-08	5.42E-07	1.67	2.16E-06	6.26E-05
132	ENSRNOG00000017078	Selenon	3.32	1.17E-08	5.52E-07	7.12	8.57E-16	2.54E-12
133	ENSRNOG00000017403	Apobr	1.32	2.35E-08	9.79E-07	1.29	3.63E-08	2.54E-06
134	ENSRNOG00000013313	Nceh1	1.30	3.29E-08	1.28E-06	1.27	5.36E-08	3.43E-06
135	ENSRNOG00000005868	Ttc21b	2.69	3.57E-08	1.36E-06	4.24	1.07E-11	3.34E-09
136	ENSRNOG00000010691	Cmtm3	1.18	4.08E-08	1.47E-06	1.18	4.78E-08	3.17E-06
137	ENSRNOG00000055295	Pecr	1.46	4.10E-08	1.47E-06	1.08	4.05E-06	1.04E-04
138	ENSRNOG00000016353	Nim1k	1.11	4.73E-08	1.69E-06	1.67	1.20E-10	2.18E-08
139	ENSRNOG00000019692	Metrn	1.84	4.95E-08	1.75E-06	1.68	2.41E-07	1.16E-05
140	ENSRNOG00000060544	Kdm5b	1.66	5.28E-08	1.82E-06	1.72	3.51E-08	2.48E-06
141	ENSRNOG00000038480	Ppp1r36	1.21	6.31E-08	2.11E-06	1.20	9.55E-08	5.31E-06
142	ENSRNOG00000004181	Sdccag8	1.37	7.35E-08	2.35E-06	1.10	1.87E-06	5.59E-05
143	ENSRNOG00000003357	Col3a1	1.59	8.31E-08	2.63E-06	3.19	4.91E-12	1.90E-09
144	ENSRNOG00000020578	Ceacam1	1.35	9.22E-08	2.83E-06	1.15	1.28E-06	4.17E-05
145	ENSRNOG00000025332	Cd109	6.49	1.11E-07	3.31E-06	9.31	4.52E-13	3.09E-10
146	ENSRNOG00000000729	Tmlhe	1.18	1.19E-07	3.49E-06	1.48	5.18E-09	4.79E-07
147	ENSRNOG00000008996	Dpysl5	1.14	1.20E-07	3.50E-06	1.28	2.80E-08	2.04E-06
148	ENSRNOG00000013862	Dusp2	5.38	1.29E-07	3.69E-06	3.38	1.89E-04	2.16E-03
149	ENSRNOG00000028632	Bcl2l14	2.41	1.34E-07	3.79E-06	1.93	6.76E-06	1.51E-04
150	ENSRNOG00000017676	Plvap	3.56	1.52E-07	4.21E-06	3.88	4.87E-08	3.20E-06
151	ENSRNOG00000014453	Anxa5	1.45	1.93E-07	5.12E-06	2.22	4.81E-10	6.90E-08
152	ENSRNOG00000016143	Gpr162	2.48	2.12E-07	5.54E-06	2.95	1.99E-08	1.51E-06
153	ENSRNOG00000033984	Ifnlr1	1.26	2.44E-07	6.17E-06	1.22	5.40E-07	2.20E-05
154	ENSRNOG00000020792	Etv4	1.78	2.85E-07	7.03E-06	1.74	5.85E-07	2.31E-05
155	ENSRNOG00000029889	Clca4	2.23	2.96E-07	7.16E-06	4.36	1.25E-12	6.93E-10
156	ENSRNOG00000003888	Rgs13	1.26	3.45E-07	8.19E-06	1.38	9.54E-08	5.31E-06
157	ENSRNOG00000059507	Lamc3	1.93	3.96E-07	9.17E-06	1.49	2.04E-05	3.72E-04
158	ENSRNOG00000009066	Thra	2.36	4.03E-07	9.30E-06	2.31	7.81E-07	2.92E-05
159	ENSRNOG00000013994	Enpp1	1.21	4.05E-07	9.30E-06	1.04	3.98E-06	1.03E-04
160	ENSRNOG00000011859	Eif5a2	1.04	8.75E-07	1.76E-05	1.15	2.65E-07	1.24E-05
161	ENSRNOG00000017857	Cul7	1.38	9.07E-07	1.80E-05	1.89	1.18E-08	9.72E-07
162	ENSRNOG00000016210	Mical2	1.92	9.21E-07	1.82E-05	2.47	2.66E-08	1.99E-06
163	ENSRNOG00000002139	Cracd	2.22	1.07E-06	2.06E-05	1.47	3.01E-04	3.10E-03
164	ENSRNOG00000000568	Slc29a3	1.33	1.13E-06	2.13E-05	1.47	3.63E-07	1.63E-05
165	ENSRNOG00000029549	Eci2	1.77	1.66E-06	2.92E-05	2.34	3.72E-08	2.57E-06
166	ENSRNOG00000036877	Clca4l	1.25	1.79E-06	3.11E-05	1.94	3.35E-09	3.17E-07
167	ENSRNOG00000028627	Hmcn1	1.49	1.85E-06	3.19E-05	1.39	6.50E-06	1.48E-04
168	ENSRNOG00000011923	Mgarp	1.92	2.09E-06	3.54E-05	1.39	1.63E-04	1.93E-03
169	ENSRNOG00000040201	Atp6ap1l	1.30	2.13E-06	3.60E-05	1.49	4.35E-07	1.88E-05
170	ENSRNOG00000020505	Map4k1	1.91	2.41E-06	3.99E-05	1.56	6.37E-05	9.18E-04
171	ENSRNOG00000021390	Meis3	1.10	3.05E-06	4.90E-05	1.31	3.36E-07	1.52E-05
172	ENSRNOG00000060867	Rln1	1.59	3.76E-06	5.77E-05	1.36	2.68E-05	4.67E-04
173	ENSRNOG00000001926	Cldn1	1.30	3.97E-06	6.03E-05	2.87	2.30E-11	5.83E-09
174	ENSRNOG00000020636	Spint2	1.22	4.02E-06	6.06E-05	1.19	5.90E-06	1.38E-04
175	ENSRNOG00000005637	Usp44	1.22	4.03E-06	6.07E-05	1.68	5.89E-08	3.69E-06
176	ENSRNOG00000013683	S1pr1	1.50	4.20E-06	6.26E-05	1.03	5.49E-04	4.87E-03
177	ENSRNOG00000026504	Fam114a1	1.61	4.61E-06	6.80E-05	2.93	1.14E-09	1.35E-07
178	ENSRNOG00000001271	Card6	1.26	5.11E-06	7.43E-05	1.19	1.17E-05	2.34E-04
179	ENSRNOG00000009225	Copz2	1.41	5.94E-06	8.33E-05	2.01	5.99E-08	3.70E-06
180	ENSRNOG00000016342	Atg10	1.55	8.70E-06	1.13E-04	1.15	3.84E-04	3.74E-03
181	ENSRNOG00000059683	Mpp2	1.47	9.88E-06	1.25E-04	1.09	3.49E-04	3.48E-03
182	ENSRNOG00000001750	Chrd	2.36	1.12E-05	1.38E-04	3.24	1.77E-07	8.85E-06
183	ENSRNOG00000014658	Zfp423	5.13	1.34E-05	1.60E-04	3.98	1.90E-03	1.25E-02
184	ENSRNOG00000016414	Slc22a17	1.13	1.44E-05	1.69E-04	1.39	1.23E-06	4.07E-05
185	ENSRNOG00000049498	Nat2	1.21	1.58E-05	1.82E-04	2.35	1.19E-09	1.39E-07
186	ENSRNOG00000033912	Fcho1	1.51	1.69E-05	1.93E-04	2.35	6.33E-08	3.83E-06
187	ENSRNOG00000018413	Per3	1.75	2.28E-05	2.42E-04	1.18	2.10E-03	1.35E-02
188	ENSRNOG00000014969	Lzts2	1.63	2.35E-05	2.47E-04	3.98	3.56E-11	7.53E-09
189	ENSRNOG00000019009	Arrdc2	1.44	2.98E-05	3.02E-04	1.54	1.98E-05	3.63E-04
190	ENSRNOG00000003400	Fmo4	1.02	2.98E-05	3.02E-04	2.06	1.73E-09	1.90E-07
191	ENSRNOG00000024501	Rgs3	1.42	3.04E-05	3.06E-04	1.79	2.08E-06	6.07E-05
192	ENSRNOG00000007462	Septin8	1.04	3.30E-05	3.28E-04	1.97	7.46E-09	6.57E-07
193	ENSRNOG00000011156	Clstn3	1.86	3.95E-05	3.80E-04	1.20	2.58E-03	1.58E-02
194	ENSRNOG00000030800	Igdcc3	2.18	6.57E-05	5.71E-04	1.44	5.51E-03	2.73E-02

	GeneID	Gene	logFC.A4vWT	PValue.A4vWT	FDR.A4vWT	logFC.D10vWT	PValue.D10vWT	FDR.D10vWT
195	ENSRNOG00000020087	Zfp90	1.21	6.70E-05	5.80E-04	1.41	1.52E-05	2.91E-04
196	ENSRNOG00000010680	Med12l	1.06	1.16E-04	9.04E-04	1.01	2.15E-04	2.38E-03
197	ENSRNOG00000001090	Stard13	1.18	1.45E-04	1.09E-03	2.96	2.44E-10	3.94E-08
198	ENSRNOG00000018452	Unc13a	1.50	1.50E-04	1.12E-03	1.60	8.41E-05	1.16E-03
199	ENSRNOG00000025121	Pla2g3	2.93	1.88E-04	1.34E-03	2.88	3.44E-04	3.45E-03
200	ENSRNOG00000013179	Tinagl1	2.00	3.05E-04	2.00E-03	2.37	5.46E-05	8.13E-04
201	ENSRNOG00000012168	Fbxl21	1.04	3.13E-04	2.05E-03	1.82	4.82E-07	2.05E-05
202	ENSRNOG00000003086	Cenpv	1.59	3.50E-04	2.25E-03	1.23	6.60E-03	3.12E-02
203	ENSRNOG00000047867	Klhdc8b	1.28	5.38E-04	3.20E-03	1.43	2.02E-04	2.27E-03
204	ENSRNOG00000006663	Usp2	1.56	7.54E-04	4.23E-03	2.58	2.00E-06	5.90E-05
205	ENSRNOG00000049976	Gzmbl3	2.34	9.37E-04	5.07E-03	2.46	7.22E-04	6.05E-03
206	ENSRNOG00000062887	Gpr35	1.01	1.35E-03	6.85E-03	1.92	1.42E-06	4.51E-05
207	ENSRNOG00000069160	Pitpnc1	1.13	1.77E-03	8.62E-03	1.15	2.28E-03	1.44E-02
208	ENSRNOG00000004438	Cntn1	1.09	1.85E-03	8.92E-03	2.60	1.09E-07	5.91E-06
209	ENSRNOG00000021966	Il17rd	1.11	2.08E-03	9.80E-03	2.21	1.13E-06	3.90E-05
210	ENSRNOG00000038102	Elmod3	1.02	4.74E-03	1.92E-02	1.20	1.70E-03	1.16E-02
211	ENSRNOG00000012630	Rhoc	1.05	7.67E-03	2.83E-02	2.44	8.51E-07	3.10E-05
212	ENSRNOG00000010121	Lef1	1.04	8.74E-03	3.14E-02	1.24	2.96E-03	1.74E-02
213	ENSRNOG00000022807	Pced1b	1.03	1.47E-02	4.74E-02	1.30	3.65E-03	2.03E-02
